# Synthesis and biological evaluation of halogenated phenoxychalcones and their corresponding pyrazolines as cytotoxic agents in human breast cancer

**DOI:** 10.1080/14756366.2021.1998023

**Published:** 2021-12-11

**Authors:** Peter A. Halim, Rasha A. Hassan, Khaled O. Mohamed, Soha O. Hassanin, Mona G. Khalil, Amr M. Abdou, Eman O. Osman

**Affiliations:** a Pharmaceutical Organic Chemistry Department, Faculty of Pharmacy, Cairo University, Cairo, Egypt; b Biochemistry Department, Faculty of Pharmacy, Modern University for Technology and Information, Cairo, Egypt; c Pharmacology and Toxicology Department, Faculty of Pharmacy, Modern University for Technology and Information, Cairo, Egypt; d Department of Microbiology and Immunology, National Research Centre, Dokki, Egypt

**Keywords:** Halogenated chalcones, diaryl ether, pyrazoline, ROS, p38 MAPK, breast cancer, cell cycle profile

## Abstract

Novel halogenated phenoxychalcones **2a–f** and their corresponding *N*-acetylpyrazolines **3a–f** were synthesised and evaluated for their anticancer activities against breast cancer cell line (MCF-7) and normal breast cell line (MCF-10a), compared with staurosporine. All compounds showed moderate to good cytotoxic activity when compared to control. Compound **2c** was the most active, with IC_50_ = 1.52 µM and selectivity index = 15.24. Also, chalcone **2f** showed significant cytotoxic activity with IC_50_ = 1.87 µM and selectivity index = 11.03. Compound **2c** decreased both total mitogen activated protein kinase (p38α MAPK) and phosphorylated enzyme in MCF-7 cells, suggesting its ability to decrease cell proliferation and survival. It also showed the ability to induce ROS in MCF-7 treated cells. Compound **2c** exhibited apoptotic behaviour in MCF-7 cells due to cell accumulation in G2/M phase and elevation in late apoptosis 57.78-fold more than control. Docking studies showed that compounds **2c** and **2f** interact with p38alpha MAPK active sites.

## 1. Introduction

There is a continuous effort in the field of synthetic medicinal chemistry to develop new molecules with better activity and safety profile^1^. Different scaffolds are continuously evaluated for their anticancer activity in the hope to overcome the side effects and improve bioavailability^2–6^. Breast cancer is one of the most common forms of invasive cancer in women. It is considered the most commonly diagnosed cancer and one of the leading causes of death among women^7^. Breast cancer is responsible for 16.2% of all female cancers and 22.9% of invasive cancers in women. It also accounts for 18.2% of all cancer deaths worldwide^8^.

Chalcones are considered as a subclass of flavonoids and they are broadly displayed in plants such as vegetables and tea^9^. They are utilised as both biosynthetic precursors of flavonoids and as end products as well^9^. Chalcones have shown different pharmacological activities including acetylcholinestrase inhibition^10^, anti-inflammatory^11^, antimicrobial^12^, antihyperlipidemic^13^, and anticancer activities^14–16^.

**Figure 1.**
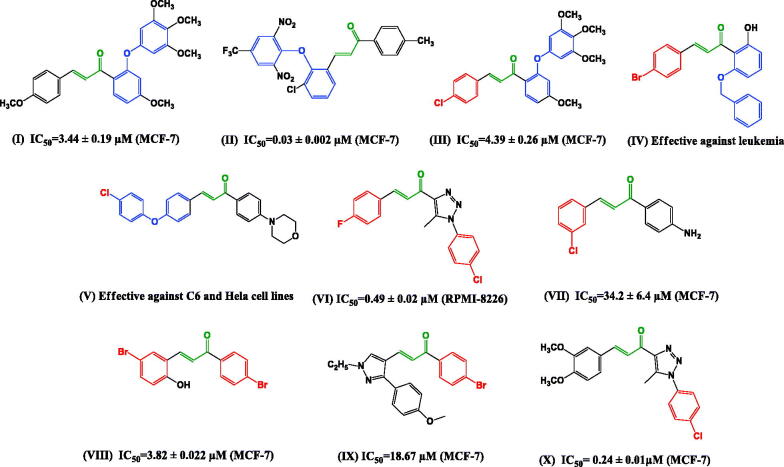
Structures of some reported diaryl ether chalcones **I–V** and halogenated chalcones **II–X** with cytotoxic activity.

The modification of chalcone scaffold to improve their bioavailability and anticancer activity has grabbed the attention of many authors. The diaryl ether scaffold is commonly found in many natural products and biologically essential molecules^17–19^. Research has shown that chalcones with a diaryl ether nucleus (**I–V**, Figure 1) possess effective cytotoxic activity including derivatives with high selectivity against breast cancer cell lines such as MCF-7 cell^20–22^. Some diaryl ether chalcones were found to express their cytotoxic activity through inhibition of tubulin polymerisation^20^^,^^21^.

Various dihalogenated chalcones have been of interest as intermediates in the synthesis of flavones and chromones^23^^,^^24^. Halogenated chalcones (**II–X**, Figure 1) have been reported to exhibit high cytotoxic activity^25–28^. They were also reported to be effective against leukaemia cell lines^15^^,^^29^ and against breast cancer cell line^15^^,^^30–32^.

Many chalcones have the ability to inhibit tumour proliferation by a variety of mechanisms as inhibition of tubulin^20^, epidermal growth factor receptor (EGFR)^33^, vascular endothelial growth factor receptor-2 (VEGFR-2)^5^^,^^15^ and mitogen-activated protein kinase (MAPK)^29^^,^^34^.

MAPKs are vital elements for intracellular transmission of signals regulating many cell functions. p38 MAPK has been demonstrated to play a major role in cell survival, proliferation, differentiation, and apoptosis in mammalian cells^35^. It was reported that the ability of chalcones to induce cell cycle arrest and apoptosis would be due to down-regulation of p38 MAPK and inhibition of its phosphorylation^36^. Targeting p38 MAPK through its inhibition or down-regulation is considered as an important strategy in the search for new anticancer molecules.

**Figure 2.**
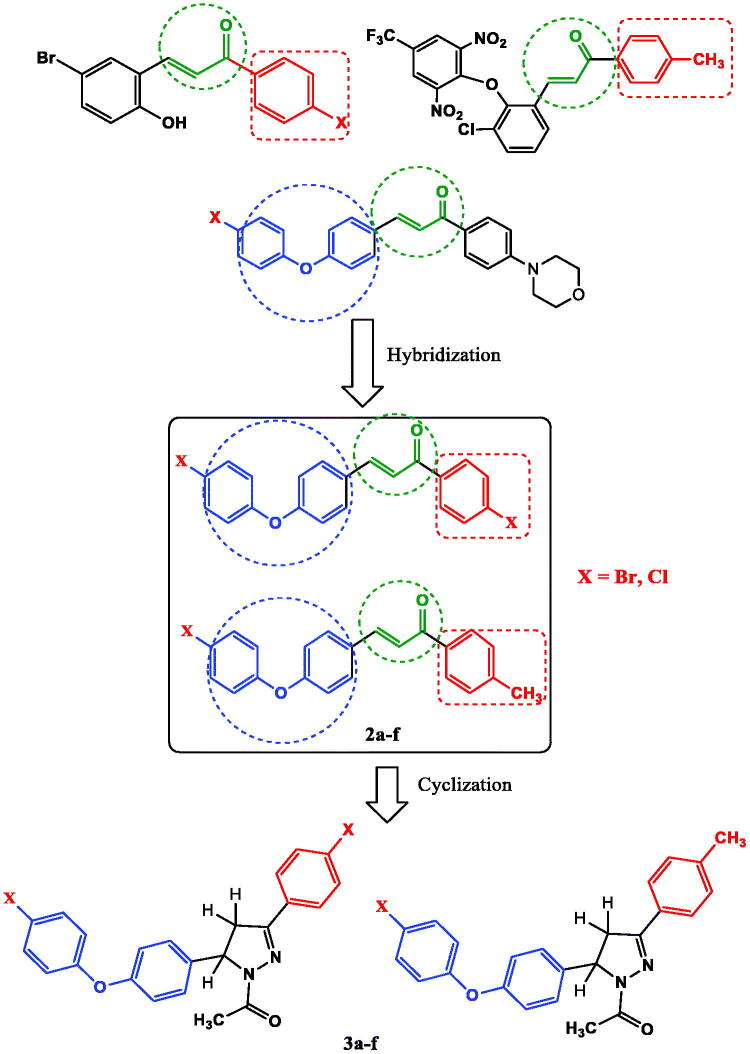
Molecular hybridisation of halogenated chalcones and diaryl ether chalcones to yield the designed chalcones **2a–f** and *N*-acetylpyrazoline derivatives **3a–f** upon cyclisation.

The objective of the current research was to design and synthesise a series of chalcones with hybridisation of halogenated chalcones and diaryl ether moiety **2a–f** (Figure 2). We planned to cyclize the synthesised chalcones to their corresponding *N*-acetylpyrazolines **3a–f** (Figure 2) as inspired by literature survey of possible anticancer activity of this scaffold^37^. We also planned to test the anticancer activity of the synthesised compounds against breast cancer.

## Materials and methods

### Chemistry

#### General

Melting points were obtained on a Griffin apparatus and were uncorrected. Microanalyses for C, H and N were carried out at the Regional Centre for Mycology and Biotechnology, Faculty of Pharmacy, Al-Azhar University. IR spectra were recorded on Shimadzu IR 435 spectrophotometer (Shimadzu Corp., Kyoto, Japan) Faculty of Pharmacy, Cairo University, Cairo, Egypt and values were represented in cm^−1^. ^1^H NMR spectra were carried out on Bruker 400 MHz (Bruker Corp., Billerica, MA, USA) spectrophotometer, Faculty of Pharmacy, Cairo University, Cairo, Egypt. Chemical shifts were recorded in ppm on *δ* scale, coupling constants (*J*) were given in Hz and peak multiplicities were designed as follows: s, singlet; d, doublet; dd, doublet of doublets; t, triplet; m, multiplet ^13^C NMR spectra were carried out on Bruker 100 MHz spectrophotometer, Faculty of Pharmacy, Cairo University, Cairo, Egypt. Progress of the reactions were monitored by TLC using precoated aluminium sheet silica gel MERCK 60 F 254 and was visualised by UV lamp. Compounds **1a,b** were previously reported in the literature^38^ and they were prepared in this work according to the reported procedure^39^.

#### General procedure for the preparation of the phenoxychalcones 2a–f

To an ice cooled, stirred solution of 4–(4-substitutedphenoxy)benzaldehyde (**1a,b**) (0.01 mol) and 4-substituted acetophenone (0.01 mol) in 95% ethanol, 40% aqueous KOH solution was added portion wise. Stirring was continued for 5 h. Solid product was filtered and washed with 3% aqueous HCl and crystallised from ethanol.

##### *3–(4-(4-Bromophenoxy)phenyl)-1–(4-chlorophenyl)prop-2-en-1-one* (2a)

Yellow solid: 86% yield; mp 150–152 °C; IR (KBr, cm^−1^) 3086 (CH aromatic),1658 (C=O), 1608 (C=C); ^1^H NMR (400 MHz, DMSO-d_6_) *δ* 8.18 (d, *J =* 8.4 Hz, 2H, ArH), 7.95 (d, *J =* 8.4 Hz, 2H, ArH), 7.88 (d, *J =* 15.7 Hz, 1H, CH=CH-CO), 7.76 (d, *J =* 15.7 Hz, 1H, CH=CH-CO), 7.66–7.60 (m, 4H, ArH), 7.11 − 7.06 (m, 4H, ArH); ^13 ^C NMR (100 MHz, DMSO-d_6_) *δ* 188.4, 158.9, 155.7, 144.2, 138.5, 136.7, 133.5, 131.6, 130.9, 130.6, 129.3, 121.9, 121.3, 119.1, 116.4; Anal. Calcd for C_21_H_14_BrClO_2_ (413.69): C, 60.97; H, 3.41, found C, 60.33; H, 3.65.

##### *3–(4-(4-Bromophenoxy)phenyl)-1–(4-bromophenyl)prop-2-en-1-one* (2b)

Yellow solid: 82% yield; mp 164–166 °C; IR (KBr, cm^−1^) 3086 (CH aromatic),1658 (C=O), 1612, (C=C); ^1^H NMR (400 MHz, DMSO-d_6_) *δ* 8.09 (d, *J =* 8.4 Hz, 2H, ArH), 7.95 (d, *J =* 8.4 Hz, 2H, ArH), 7.87 (d, *J =* 15.6 Hz, 1H, CH=CH-CO), 7.80–7.74 (m, 3H, 2ArH + CH=CH-CO), 7.61 (d, *J =* 8.4 Hz, 2H, ArH), 7.11–7.06 (m, 4H, ArH); ^13^C NMR (100 MHz, DMSO-d_6_) *δ* 188.7, 159.0, 155.7, 144.3, 137.1, 133.5, 132.3, 131.6, 131.0, 130.6, 127.7, 121.9, 121.3, 119.1, 116.4: Anal. Calcd for C_21_H_14_Br_2_O_2_ (458.14): C, 55.05; H, 3.08, found C, 54.81 H, 3.52.

##### *3–(4-(4-Bromophenoxy)phenyl)-1-(p-tolyl)prop-2-en-1-one* (2c)

Yellow solid: 90% yield; mp 157–159 °C; IR (KBr, cm^−1^) 3089 (CH aromatic),1658 (C=O), 1600, (C=C); ^1^H NMR (400 MHz, DMSO-d_6_) *δ* 8.07 (d, *J =* 8.0 Hz, 2H, ArH), 7.94 (d, *J =* 8.4 Hz, 2H, ArH), 7.87 (d, *J =* 15.6 Hz, 1H, CH=CH-CO), 7.73 (d, *J =* 15.6 Hz, 1H, CH=CH-CO), 7.60 (d, *J =* 8.4 Hz 2H, ArH), 7.38 (d, *J =* 8.0 Hz 2H, ArH), 7.10–7.05 (m, 4H, ArH), 2.41(s, 3H, CH_3_);^13^C NMR (100 MHz, DMSO-d_6_) *δ* 189.0, 158.7, 155.8, 144.0, 143.3, 135.6, 133.4, 131.5, 130.8, 129.8, 129.1, 121.8, 121.7, 119.1, 116.3, 21.7; Anal. Calcd for C_22_H_17_BrO_2_ (393.27): C, 67.19; H, 4.36, found C, 67.64; H, 4.43.

##### *3–(4-(4-Chlorophenoxy)phenyl)-1–(4-chlorophenyl)prop-2-en-1-one* (2d)

Yellow solid: 83% yield; mp 120–122 °C; IR (KBr, cm^−1^) 3089 (CH aromatic), 1658 (C=O), 1608, (C=C); ^1^H NMR (400 MHz, DMSO-d_6_) *δ* 8.18 (d, *J =* 8.4 Hz, 2H, ArH), 7.95 (d, *J =* 8.4 Hz, 2H, ArH), 7.88 (d, *J =* 15.6 Hz, 1H, CH=CH-CO), 7.76 (d, *J =* 15.6 Hz, 1H, CH=CH-CO), 7.65 (d, *J =* 8.4 Hz, 2H, ArH), 7.48 (d, *J =* 8.4 Hz, 2H, ArH), 7.14–7.08 (m, 4H, ArH); ^13^C NMR (100 MHz, DMSO-d_6_) *δ* 188.4, 159.1, 155.1, 144.2, 138.5, 136.7, 131.6, 130.8, 130.5, 130.5, 129.3, 128.5, 121.5, 121.2, 118.9; Anal. Calcd for C_21_H_14_Cl_2_O_2_ (369.24): C, 68.31; H, 3.82, found C, 67.72; H, 4.11**.**

##### *1–(4-Bromophenyl)-3–(4-(4-chlorophenoxy)phenyl)prop-2-en-1-one* (2e)

Yellow solid: 79% yield; mp 143–145 °C; IR (KBr, cm^−1^) 3089 (CH aromatic), 1658 (C=O), 1608, (C=C); ^1^H NMR (400 MHz, DMSO-d_6_) *δ* 8.09 (d, *J =* 8.4 Hz, 2H, ArH), 7.94 (d, *J =* 8.4 Hz, 2H, ArH), 7.87 (d, *J =* 15.6 Hz, 1H, CH=CH-CO), 7.80– 7.74 (m, 3H, ArH, CH=CH-CO), 7.48 (d, *J =* 8.4 Hz, 2H, ArH), 7.14–7.08 (m, 4H, ArH); ^13^C NMR (100 MHz, DMSO-d_6_) *δ* 188.7, 159.1, 155.1, 144.3, 137.1, 132.3, 131.6, 131.0, 130.5, 130.5, 128.5, 127.7, 121.5, 121.2, 118.9; Anal. Calcd for C_21_H_14_BrClO_2_ (413.69): C, 60.97; H, 3.41, found C, 60.63; H, 3.78.

##### *3–(4-(4-Chlorophenoxy)phenyl)-1-(p-tolyl)prop-2-en-1-one* (2f)

Yellow solid: 86% yield; mp 136–138 °C; IR (KBr, cm^−1^) 3089 (CH aromatic),1658 (C=O), 1600 (C=C); ^1^H NMR (400 MHz, DMSO-d_6_) *δ* 8.07 (d, *J =* 8.0 Hz, 2H, ArH), 7.93 (d, *J =* 8.0 Hz, 2H, ArH), 7.87 (d, *J =* 15.6 Hz, 1H, CH=CH-CO), 7.73 (d, *J =* 15.6 Hz, 1H, CH=CH-CO), 7.48 (d, *J =* 8.0 Hz, 2H, ArH), 7.38 (d, *J =* 8.0 Hz, 2H, ArH), 7.14– 7.07 (m, 4H, ArH), 2.41 (s, 3H, CH_3_); ^13^C NMR (100 MHz, DMSO-d_6_) *δ*:189.0, 158.9, 155.2, 144.0, 143.3, 135.6, 131.4, 130.7, 130.5, 129.8, 129.1, 128.4, 121.7, 121.4, 119.0, 21.7; Anal. Calcd for C_22_H_17_ClO_2_ (348.82): C, 75.75; H, 4.91, found C, 76.08; H, 4.68.

#### *General procedure for the preparation of* *substituted 4,5-dihydro-1H-pyrazol-1-yl)ethan-1-one* *(3a–f)*

A mixture of phenoxychalcones **2a–f** (1.0 mmol), hydrazine hydrate (5.0 mmol) and acetic acid (5.0 ml) was heated under reflux for 3 h. The reaction mixture was cooled and poured onto crushed ice. Solid product was filtered, washed with water and crystallised from ethanol.

##### 1–(5-(4–(4-Bromophenoxy)phenyl)-3–(4-chlorophenyl)-4,5-dihydro-1H-pyrazol-1-yl) ethanone (3a)

White solid: 89% yield; mp 142–144 °C; IR (KBr, cm^−1^) 3051 (CH aromatic), 2943, 2927 (CH aliphatic), 1658 (C=O), 1593 (C=N); ^1^H NMR (400 MHz, DMSO-d_6_) *δ* 7.80 (d, *J =* 8.0 Hz, 2H, ArH), 7.55– 7.52 (m, 4H, ArH), 7.23 (d, *J =* 8.0 Hz, 2H, ArH), 6.99 (d, *J =* 8.4 Hz, 2H, ArH), 6.94 (d, *J =* 8.4 Hz, 2H, ArH), 5.56 (dd, *J =* 12.0, 4.4 Hz, 1H, H_X_ pyrazoline), 3.85 (dd, *J =* 18.4, 12.0 Hz, 1H, H_B_ pyrazoline), 3.16 (dd, *J =* 18.4, 4.4 Hz, 1H, H_A_ pyrazoline), 2.31 (s, 3H, CH_3_CO); ^13^C NMR (100 MHz, DMSO-d_6_) *δ* 168.1, 156.6, 155.6, 153.7, 138.3, 135.4, 133.2, 130.4, 129.3, 128.8, 127.8, 121.0, 119.5, 115.5, 59.6, 42.4, 22.2; Anal. Calcd for C_23_H_18_BrClN_2_O_2_ (469.76): C, 58.81; H, 3.86; N, 5.96, found C, 58.57; H, 3.61; N, 6.12.

##### *1–(5-(4–(4-Bromophenoxy)phenyl)-3–(4-bromophenyl)-4,5-dihydro-1H-pyrazol-1-yl) ethanone* (3b)

White solid: 85% yield; mp 138–140 °C; IR (KBr, cm^−1^) 3051 (CH aromatic),2974, 2924 (CH aliphatic), 1658 (C=O), 1589 (C=N); ^1^H NMR (400 MHz, DMSO-d_6_) *δ* 7.73 (d, *J =* 8.4 Hz, 2H, ArH), 7.67 (d, *J =* 8.4 Hz, 2H, ArH), 7.54 (d, *J =* 8.4 Hz, 2H, ArH), 7.22 (d, *J =* 8.4 Hz, 2H, ArH), 7.00–6.94 (m, 4H, ArH), 5.56 (dd, *J =* 11.8, 4.6 Hz, 1H, H_X_ pyrazoline), 3.85 (dd, *J =* 18.2, 11.8 Hz, 1H, H_B_ pyrazoline), 3.16 (dd, *J =* 18.2, 4.6, Hz, 1H, H_A_ pyrazoline), 2.31 (s, 3H, CH_3_CO); ^13^C NMR (100 MHz, DMSO-d_6_) *δ* 168.0, 156.6, 155.6, 153.8, 138.3, 133.2, 132.2, 130.8, 129.0, 127.9, 124.1, 121.0, 119.5, 115.5, 59.6, 42.4, 22.2; Anal. Calcd for C_23_H_18_Br_2_N_2_O_2_ (514.21): C, 53.72; H, 3.53; N, 5.45, found C, 53.51; H, 3.28; N, 5.69.

##### *1–(5-(4–(4-Bromophenoxy)phenyl)-3-(p-tolyl)-4,5-dihydro-1H-pyrazol-1-yl)ethanone* (3c)

White solid: 92% yield; mp 141–143 °C; IR (KBr, cm^−1^) 3050 (CH aromatic), 2947, 2920 (CH aliphatic), 1662 (C=O), 1585 (C=N); ^1^H NMR (400 MHz, DMSO-d_6_) *δ* 7.68 (d, *J =* 8.0 Hz, 2H, ArH), 7.54 (d, *J =* 8.4 Hz, 2H, ArH), 7.28 (d, *J =* 8.0 Hz, 2H, ArH), 7.22 (d, *J =* 8.4 Hz, 2H, ArH), 6.99 (d, *J =* 8.4 Hz, 2H, ArH), 6.95 (d, *J =* 8.4 Hz, 2H, ArH), 5.54 (dd, *J =* 12.0, 4.4 Hz, 1H, H_X_ pyrazoline), 3.83 (dd, *J =* 18.0, 12.0 Hz, 1H, H_B_ pyrazoline), 3.12 (dd, *J =* 18.0, 4.4 Hz, 1H, H_A_ pyrazoline), 2.35 (s, 3H, CH_3_CO), 2.30 (s, 3H, CH_3_); ^13^C NMR (100 MHz, DMSO-d_6_) *δ* 167.8, 156.6, 155.6, 154.6, 140.6, 138.5, 133.2, 129.8, 128.8, 127.8, 127.1, 121.0, 119.5, 115.5, 59.2, 42.6, 22.2, 21.5; Anal. Calcd for C_24_H_21_BrN_2_O_2_ (449.34): C, 64.15; H, 4.71; N, 6.23, found C, 64.43; H, 4.62; N, 6.34.

##### 1–(5-(4–(4-Chlorophenoxy)phenyl)-3–(4-chlorophenyl)-4,5-dihydro-1H-pyrazol-1-yl) ethanone (3d)

White solid: 81% yield; mp 116–118 °C; IR (KBr, cm^−1^) 3043 (CH aromatic), 2978, 2924 (CH aliphatic), 1658 (C=O), 1593 (C=N); ^1^H NMR (400 MHz, DMSO-d_6_) *δ* 7.81 (d, *J* = 8.4 Hz, 2H, ArH), 7.54 (d, *J* = 8.4 Hz, 2H, ArH), 7.42 (d, *J* = 8.4 Hz, 2H, ArH), 7.22 (d, *J* = 8.4 Hz, 2H, ArH), 7.02– 6.98 (m, 4H, ArH), 5.56 (dd, *J* = 12.0, 4.4 Hz, 1H, H_X_ pyrazoline), 3.85 (dd, *J* = 18.0, 12.0 Hz, 1H, H_B_ pyrazoline), 3.16 (dd, *J =* 18.0, 4.4 Hz, 1H, H_A_ pyrazoline), 2.31 (s, 3H, CH_3_CO); ^13^C NMR (100 MHz, DMSO-d_6_) *δ* 168.0, 156.1, 155.8, 153.7, 138.3, 135.3, 130.5, 130.3, 129.3, 128.8, 127.9, 127.6, 120.6, 119.4, 59.6, 42.4, 22.2;Anal. Calcd for C_23_H_18_Cl_2_N_2_O_2_ (425.31): C, 64.95; H, 4.27; N, 6.59, found C, 65.18; H, 4.02; N, 6.87

##### *1–(3-(4-Bromophenyl)-5–(4-(4-chlorophenoxy)phenyl)-4,5-dihydro-1H-pyrazol-1-yl) ethanone* (3e)

White solid: 80% yield; mp 117–119 °C; IR (KBr, cm^−1^) 3050 (CH aromatic), 2945, 2920 (CH aliphatic), 1658 (C=O), 1585 (C=N); ^1^H NMR (400 MHz, DMSO-d_6_) *δ* 7.73 (d, *J* = 8.4 Hz, 2H, ArH), 7.67 (d, *J* = 8.8 Hz, 2H, ArH), 7.42 (d, *J* = 8.8 Hz, 2H, ArH), 7.22 (d, *J* = 8.8 Hz, 2H, ArH), 7.02–6.98 (m, 4H, ArH), 5.56 (dd, *J* = 12.0, 4.4 Hz, 1H, H_X_ pyrazoline), 3.85 (dd, *J* = 18.0, 12.0 Hz, 1H, H_B_ pyrazoline), 3.16 (dd, *J* = 18.0. 4.4 Hz, 1H, H_A_ pyrazoline), 2.31 (s, 3H, CH_3_CO); ^13^C NMR (100 MHz, DMSO-d_6_) *δ* 168.0, 156.1, 155.8, 153.8, 138.3, 132.2, 130.8, 130.3, 129.0, 127.9, 127.6, 124.1, 120.6, 119.4, 59.6, 42.4, 22.2; Anal. Calcd for C_23_H_18_BrClN_2_O_2_ (469.76): C, 58.81; H, 3.86; N, 5.96, found C, 59.13; H, 3.55; N, 5.73.

##### *1–(5-(4–(4-Chlorophenoxy)phenyl)-3-(p-tolyl)-4,5-dihydro-1H-pyrazol-1-yl) ethanone* (3f)

Yellow solid: 88% yield; mp 118–120 °C; IR (KBr, cm^−1^) 3039 (CH aromatic), 2947, 2920 (CH aliphatic), 1658 (C=O), 1589 (C=N); ^1^H NMR (400 MHz, DMSO-d_6_) *δ* 7.69 (d, *J* = 8.0 Hz, 2H, ArH), 7.42 (d, *J* = 8.8 Hz, 2H, ArH), 7.28 (d, *J* = 8.0 Hz, 2H, ArH), 7.22 (d, *J =* 8.8 Hz, 2H, ArH), 7.02–6.98 (m, 4H, ArH), 5.54 (dd, *J =* 12.0, 4.4 Hz, 1H, H_X_ pyrazoline), 3.84 (dd, *J =* 18.0, 12.0 Hz, 1H, H_B_ pyrazoline), 3.13 (dd, *J =* 18.0. 4.4 Hz, 1H, H_A_ pyrazoline), 2.35 (s, 3H, CH_3_CO), 2.30 (s, 3H, CH_3_); ^13^C NMR (100 MHz, DMSO-d_6_) *δ* 167.8, 156.1, 155.7, 154.6, 140.6, 138.4, 130.3, 129.8, 128.8, 127.8, 127.6, 127.1, 120.6, 119.4, 59.2, 42.6, 22.2, 21.5; Anal. Calcd for C_24_H_21_ClN_2_O_2_ (404.89): C, 71.19; H, 5.23; N, 6.92, found C, 71.45; H, 5.56; N, 6.61.

## Biological evaluation

### Chemicals

#### General anticancer assessment using MTT assay

##### Cell Culture

Human breast cancer cell line MCF-7 and normal breast cell line MCF-10a were obtained from American type culture collection, MCF-7 cells were cultured using Dulbecco’s Modified Eagle Medium DMEM (Invitrogen/Life Technologies) supplemented with 10% FBS (Hyclone), 10 µg/ml of insulin (Sigma), and 1% penicillin-streptomycin. MCF-10a cell line was cultured using DMEM medium supplemented with 10% FBS, 1% sodium pyruvate, 1% MEM NEAA, 20 ng/ml human epidermal growth factor (HEGF), 10 μg/ml insulin and 0.05 mM hydrocortisone. The cells were incubated at 37 °C in a humidified atmosphere of 5% CO_2._

#### MTT assay

The anticancer effect of phenoxychalcones and *N*-acetylpyrazolines on the viability of both tumour and normal cells was evaluated using МТТ assay as a rapid colorimetric assay. It is based on the ability of mitochondrial dehydrogenases of viable cells to reduce yellow tetrazolium salt MTT to blue formazan in quantities proportional to the number of living cells^40^. The cells were incubated with the medium alone or with the tested compounds at concentrations of 100 µM, 25 µM, 6.25 µM, 1.56 µM and 0.39 µM for 48 h. Staurosporine tested for 48 h of incubation served as positive control. Then, 20 μL MTT solution was added to each well and mixed. After 4 h, the supernatants were removed and 100 μL DMSO was added to each well to dissolve the precipitate. The viability of cells was estimated by measuring the absorbance at 570 nm using a Bioline ELIZA plate reader. The cell viability percentage was calculated based on the absorbance ratio between cell culture treated with the compounds and the untreated control multiplied by 100. Selectivity index (SI) was calculated as the ratio of cytotoxicity (IC_50_) on normal cells (MCF-10a) to cancer cells (MCF-7).

#### Cell cycle analysis

The cell cycle analysis was carried out by flow cytometry using ab139418_propidium iodide flow cytometry kit/BD (Abcam, Cambridge, UK) according to the manufacturer instructions. Briefly, MCF-7 cells were treated with compound **2c** at its IC_50_ concentration for 24 h. After treatment, the cells were washed twice with ice-cold phosphate buffer saline (PBS) and collected by centrifugation. The cells were then fixed using ice-cold 66% (v/v) ethanol, washed with PBS, and re-suspended with 0.1 mg/mL RNase to digest cellular RNA and thus minimise stained RNA in the background. The cells were then stained with 40 mg/mL propidium iodide (PI), a fluorescent molecule with the ability to bind to nucleic acid. PI binds to the DNA in cells in proportion to its amount. As the DNA content is different among cells in different phases of cell cycle, the stage of cell growth can be determined based on fluorescence intensity. Cell fluorescence was evaluated using FacsCalibur (BD Biosciences, USA) and analysed using Cell- Quest software (Becton Dickinson)^41^. Cell cycle analysis of MCF-7 cells without any treatment was used as control.

#### Detection of apoptosis

Apoptosis assessment was carried out by flow cytometry using Annexin V-FITC and propidium iodide double-staining apoptosis detection kit (Biovision, USA) according to the manufacturer instructions. Annexin V is a protein with high affinity for phosphatidylserine (PS). The latter is a cell membrane component that translocate from the inner face of the plasma membrane to the cell surface soon after initiating apoptosis. Once on the cell surface, PS can be detected by fluorescent conjugate of Annexin V. Briefly, 5 × 10^5^ MCF-7 cells were treated with compound **2c** at its IC_50_ concentration for 24 h. The cells were then collected by centrifugation and re-suspended in 500 μL of binding buffer. Annexin V-FITC and PI double staining was performed by adding 5 μL of Annexin V-FITC and 5 μL of PI. The cells were then incubated in dark, at room temperature for 5 min. After incubation, cell fluorescence was evaluated using FacsCalibur (BD Biosciences, USA). Dot-plot graphs were used to illustrate the results.

#### Intracellular reactive oxygen species (ROS) analysis

Intracellular ROS was detected by cell-permeable fluorogenic probe H2DCFDA. Cell culture subdivided into three groups. The groups are the negative control (untreated MCF-7 cells), the positive control group (MCF-7 cells treated with H_2_O_2_), and test group (MCF-7 cells treated with compound **2c**). After removing the culture supernatants, the cells were then incubated in fresh medium with 10 µM H2DCFDA at the dark at 37 ^o^C for 1 h. The cells were washed with PBS buffer, then, harvested and the pellets were suspended in PBS. Samples were analysed ROBONIK P2000 ELISA reader wavelength 450 nm.

#### p38 MAPK (phosphorylated and total)

Measurements were performed using ELISA kits (R&D systems, Minneapolis, MN, USA ELISA (Cat. # KHO0071) and p38 MAPK (Total) ELISA, according to the manufacturer’s instructions.

#### Molecular docking in the active site of p38alpha MAP kinase

All the molecular modelling studies were carried out using Molecular Operating Environment (MOE, 2019.0102) software. All minimizations were performed with MOE until an RMSD gradient of 0.1 kcal·mol^−1 ^Å^−1^ with MMFF94x force field and the partial charges were automatically calculated. The X-ray crystallographic structure of p38alpha MAP Kinase complexed with [5-amino-1–(4-fluorophenyl)-1H-pyrazol-4-yl][3-(piperidin-4-yloxy)phenyl]methanone (PQA) ligand (**PDB ID: 2BAL**)^42^ was downloaded from the protein data bank^43^. For each co-crystallised enzyme; water molecules and ligands which are not involved in the binding were removed, the protein was prepared for the docking study using *Protonate 3 D* protocol in MOE with default options. The co-crystalized ligand (PQA) was used to define the binding site for docking. Triangle matcher placement method and London dG scoring function were used for docking.

### Statistical analysis

Data are represented as mean ± SD. Significant differences between groups were analysed by using one-way ANOVA with post-hoc comparisons using Tukey’s test (SPSS v18, Chicago, IL, USA). Differences were considered significant at *p* < 0.05.

## Results and discussion

### Chemistry

**Scheme 1.**
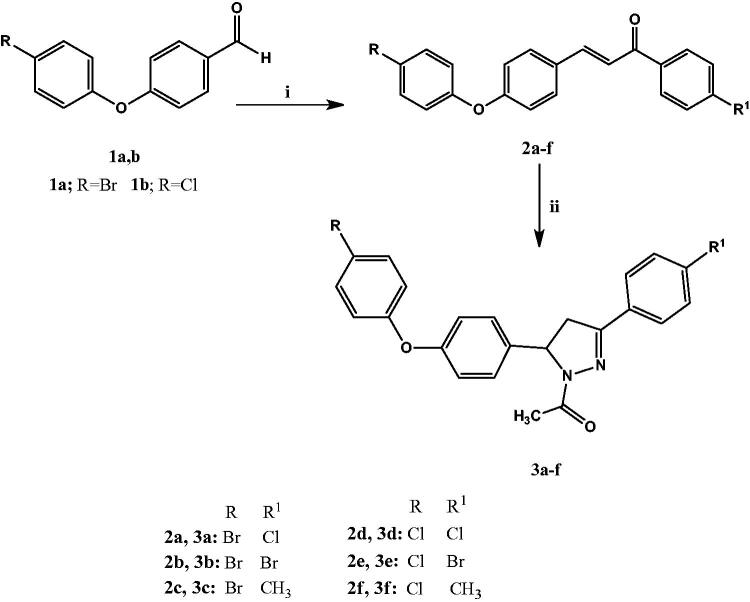
Synthesis of phenoxy halogenated chalcones **2a–f** and *N*-acetylpyrazolines **3a–f**. Reagents and conditions: (i) *p*-Substituted acetophenone, 40% KOH, 95% ethanol, 0 °C, (ii) Hydrazine hydrate, GAA, reflux 3 h.

Novel halogenated phenoxychalcones **2a–f** and their corresponding *N*-acetylpyrazolines **3a–f** were synthesised according to Scheme 1. Phenoxybenzaldehydes were previously prepared by reacting 4-nitrobenzaldehyde and phenylboronic acid using rhodium catalyst and CsCO_3_ as a base^38^. However, in this research phenoxybenzaldydes **1a**,**b** were prepared via Williamson etherification of 4-substituted phenols and 4-fluorobenzaldehyde according to a reported method^39^. A series of 1,3-diaryl-2-propen-l-ones **2a–f** was obtained via reacting compounds **1a**,**b** with different 4-substituted acetophenones under base catalysed Claisen–Schmidt condensation^44^^,^^45^ in a good to excellent yield (79–90%). The structures of compounds **2a–f** were confirmed using different spectroscopic methods. IR spectra characteristically revealed the existence of peaks for conjugated carbonyl groups at 1658 cm^−1^. ^1^H NMR spectra obviously showed two doublet signals corresponding to the vinyl protons CH = CH–CO with coupling constant 15.6–15.7 Hz indicating *trans* configuration^46^^,^^47^. Also, ^13^C NMR spectra showed the presence of carbonyl carbon at *δ* 188.4–188.9 ppm along with *trans* olefinic carbons at *δ* 144.2–143.9 and *δ* 121.2–121.6 ppm.

**Figure 3.**
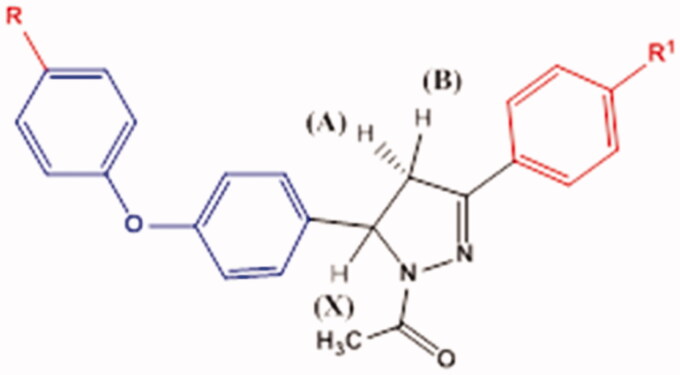
ABX spin system on the pyrazoline derivatives **3a–f**.

A novel series of *N*-acetylpyrazolines **3a–f** was prepared in excellent yield (81–92%) by cyclocondensation of phenoxy halogenated chalcones **2a–f** using hydrazine hydrate in acetic acid as solvent^48^. The characterisation of **3a–f** was conducted by IR spectra showing the presence of aliphatic CH at 2920–2978 cm^−1^ and C=N peaks at 1589–1593 cm^−1^, as well as disappearance of carbonyl group signals thus indicating the formation of a pyrazoline ring. The pyrazoline ring has a stereogenic carbon, thus compounds **3a–f** exist in two stereoisomers R and S. This was obviously revealed by ^1^H NMR spectra which verified the assigned structures of **3a–f** and showed clear characteristic signals of ABX spin system on the pyrazoline ring (Figure 3)^49^^,^^50^. The two geminal protons of the pyrazoline ring resonated as two doublet of doublets at *δ* 3.12–3.16 ppm due to H_A_ of pyrazoline ring and at *δ* 3.83–3.85 ppm due to H_B_ of pyrazoline with geminal coupling constant of 18.0–18.4 Hz. On the other hand, the vicinal H_X_ pyrazoline proton appeared as a doublet of doublet at *δ* 5.54–5.56 ppm due to coupling with two magnetically non-equivalent protons with coupling constant of 4.4–4.6 Hz due to coupling with *trans* H_A_ and 11.8–12.0 Hz due to coupling with *cis* H_B_. The CH_3_ protons of the acetyl group were observed at *δ* 2.31–2.35 ppm in ^1^H NMR spectra. ^13^C NMR spectra offered further evidence for *N*-acetylpyrazoline structure indicating the absence of trans-olefinic carbon signals and the presence of signals at *δ* 59.2–59.5 ppm corresponding for C5 pyrazoline and *δ* 42.3–42.5 ppm due to C4 of pyrazoline, in addition to a signal at *δ* 22.1–22.2 ppm due to CH_3_ of the acetyl group.

## Biological evaluation

### Evaluation of anticancer activity

The synthesised chalcones **2a–f** and their corresponding *N*-acetylpyrazolines **3a–f** were evaluated for their anticancer activity against breast cancer cell line (MCF-7) and normal breast cell line (MCF-10a) using MTT assay protocol. The inhibitory concentration that exhibited 50% cell death (IC_50_) was calculated for each compound as well as selectivity index for cancer cell line over normal cells. Staurosporine was used as reference drug. It is a kinase inhibitor and a strong inducer of apoptosis^51^. It was also reported as death inducer to MCF-7 human breast cancer cells^52^^,^^53^. The results were displayed in Table 1.

**Table 1.** *In vitro* anticancer screening against cancer cell line MCF-7 and normal cell line MCF-10a compared to staurosporine.

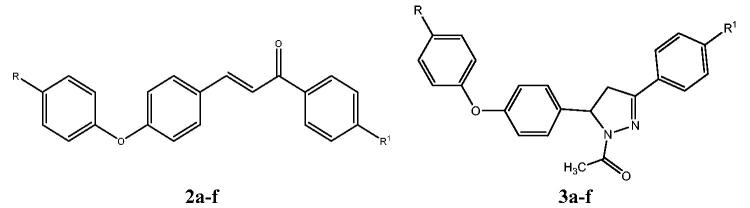					
			*In vitro* cytotoxicity IC_50_ (μM)^a^		
Compound	R	R^1^	MCF-7	MCF-10a	Selectivity index^b^
**2a**	Br	Cl	8.51 ± 0.16	33.39 ± 1.34	3.92
**2b**	Br	Br	13.28 ± 0.53	25.33 ± 1.17	1.91
**2c**	Br	CH_3_	1.52 ± 0.03	23.16 ± 0.88	15.24
**2d**	Cl	Cl	19.87 ± 0.66	33.15 ± 1.42	1.67
**2e**	Cl	Br	44.20 ± 2.16	22.12 ± 1.08	0.50
**2f**	Cl	CH_3_	1.87 ± 0.02	20.63 ± 0.37	11.03
**3a**	Br	Cl	10.56 ± 0.39	55.82 ± 1.92	5.29
**3b**	Br	Br	17.17 ± 0.62	11.54 ± 0.47	0.67
**3c**	Br	CH_3_	7.42 ± 0.28	19.52 ± 0.69	2.63
**3d**	Cl	Cl	8.51 ± 0.27	45.74 ± 1.36	5.37
**3e**	Cl	Br	5.02 ± 0.22	10.12 ± 0.62	2.02
**3f**	Cl	CH_3_	4.77 ± 0.13	12.16 ± 0.79	2.55
**Staurosporine**			5.90 ± 0.14	15.33 ± 0.51	2.60

^a^IC_50_ values are the mean ± SD of three separate experiments.

^b^Selectivity index was calculated as the ratio of cytotoxicity (IC_50_) on normal cells (MCF-10a) to cancer cells (MCF-7).

**Figure 4.**
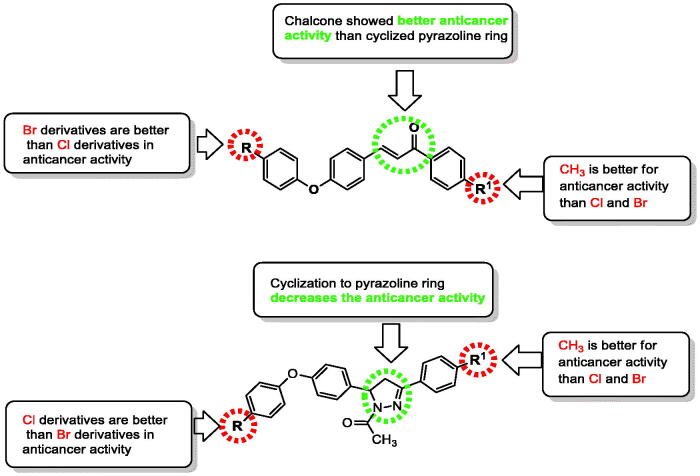
Structure activity relationship (SAR) of the synthesised chalcones **2a–f** and their cyclized derivatives *N*-acetylpyrazolines **3a–f**.

In a closer look at Table 1, the IC_50_ values on MCF-7 for the compounds can be used to explore structure activity relationship of the synthesised chalcones **2a–f** and their corresponding pyrazolines **3a–f** using staurosporine as reference drug (Figure 4).

For chalcone derivatives **2a–f**: the "R" substitution with bromo in the para position of diaryl ether moiety **2a–c** exhibited better activity than their chloro analogues **2d–f**. The IC_50_ for 4-bromo-phenoxy derivatives ranged between 1.52–13.28 µM with superior selectivity for compound **2c** of 15.24. On the other hand, 4-chlorophenoxy derivatives exhibited IC_50_ values in the range of 1.87–44.20 µM with selectivity index range of 0.5–11.03. What was noticed in this group of compounds is the same pattern of activity for substitutions in para position of the phenyl ring as derivatives with R^1^ = CH_3_ were the most active compounds **2c** and **2f** with IC_50_ 1.52 and 1.87 µM, respectively. The second place was occupied by the derivatives **2a** and **2d** having R^1^ = Cl, while the least active derivatives were **2 b** and **2e** with R^1^ = Br (Figure 4).

For pyrazoline derivatives **3a–f**, phenoxypyrazolines **3d–f** with R = Cl was more active than phenoxypyrazolines **3a–c** with R = Br. The IC_50_ for 4-chlorophenoxypyrazoline derivatives **3d–f** ranged between 4.77–8.51 µM with selectivity index range of 2.02–5.37. On the other hand, bromophenoxy derivatives **3a–c** exhibited IC_50_ values in the range of 7.42–17.17 µM with the existence of non-selective compound **3b,** with selectivity index of 0.67. Interestingly, the same pattern of activity for substitutions in para position of the phenyl ring that was noticed in chalcone derivatives was repeated in bromophenoxypyrazolines where R^1^ = CH_3_ **3c** was the most active compound with IC_50_ 7.42 µM followed by derivatives with R^1^ = Cl then derivatives with R^1^ = Br. This pattern changed in one ranking position for 4-chlorophenoxy pyrazolines **3d–f** where the most active compound was again the derivative having R^1^ = CH_3_ **3f** with IC_50_ 4.77 µM followed by bromo analogue this time and finally the chloro derivative. Literature survey revealed that, as pyrazoline ring is chiral, the activity of some biologically important pyrazoline compounds is due to one of the isomers, either R or S^54^. However, in this study, pyrazoline derivatives **3a–f** were not as biologically interesting as the chalcone derivatives **2a–f**, thus no isomer separation was needed.

As an important point to discuss α,β-unsaturated compounds, as Michael acceptors, are of special interest due to their ability to undergo irreversible covalent interaction with MAPK leading to its inhibition^55^. However, the possibility of being pan assay interference compounds (PAINS) may be an obstacle to many researchers. PAINS are considered as useless compounds in the drug discovery process as they show activity in multiple types of assays^56^^,^^57^. However, in this study, it was found that the most active chalcone **2c** with methyl substituent at R^1^ was 5.6-fold and 8.7-fold more active than its bioisosters **2a** and **2b,** respectively. Also chalcone **2f** with methyl substituent at R^1^ was 10.6-fold and 23.6-fold more active than chalcones **2d** and **2e,** respectively. Moreover, the high selectivity index of compounds **2c** and **2f** excluded the doubt of being PAINS.

### Cell cycle analysis

**Figure 5.**
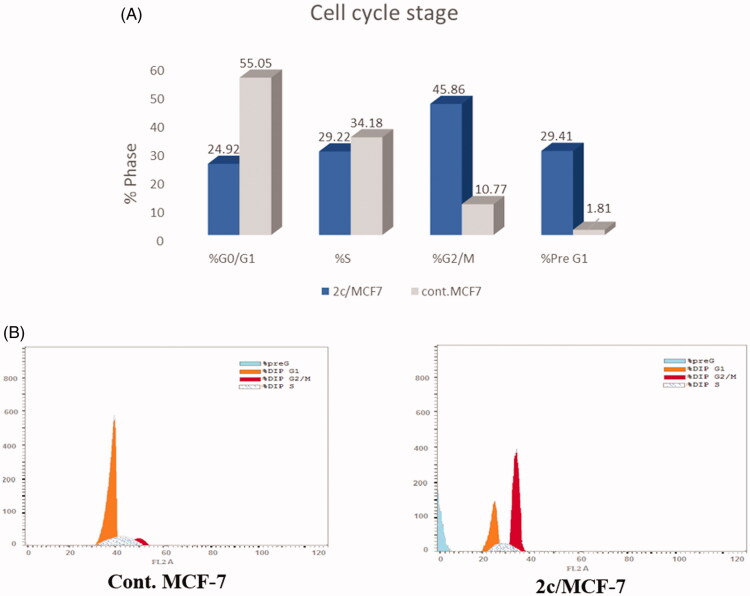
(A) Graphical representation of the effect of compound **2c** on the cell cycle stages of MFC-7 cells. (B) Effect of compound **2c** (1.52 µM) on DNA-ploidy flow cytometric analysis of MFC-7 cells after 24 h.

Anticancer agents exert their cytotoxic action by terminating cellular proliferation at definite checkpoints found at different stages of the cell cycle. Suppression of these phases results in the termination of the cell proliferation. Cell cycle analysis employs flow cytometry to differentiate between cells within different phases of the cell cycle. In this work, the effect of the most active compound **2c** on cell cycle progression was studied to explore the definite phase at which cell cycle arrest takes place in the MCF-7 breast cancer cell line. MCF-7 cells were treated with compound **2c** at its IC_50_ concentration (1.52 μM) and its effect on the cell population in different cell phases was recorded and displayed in Figure 5. Exposure of MCF-7 cells to compound **2c** resulted in significant decline in the cell population at the G0/G1 and S phases with 54.73% (from 55.05% to 24.92%) and 14.5% (from 34.18% to 29.22%), respectively. Moreover, marked augmentation was observed in the proportion of cells in the G2/M phase by 4.25-fold, and in the pre-G1 phase by 16.24-fold, in comparison to the control (DMSO). This clearly indicates that the target chalcone derivative **2c** arrested the cell cycle proliferation of MCF-7 cells in the G2/M phase.

### Annexin V-FITC apoptosis assay

**Figure 6.**
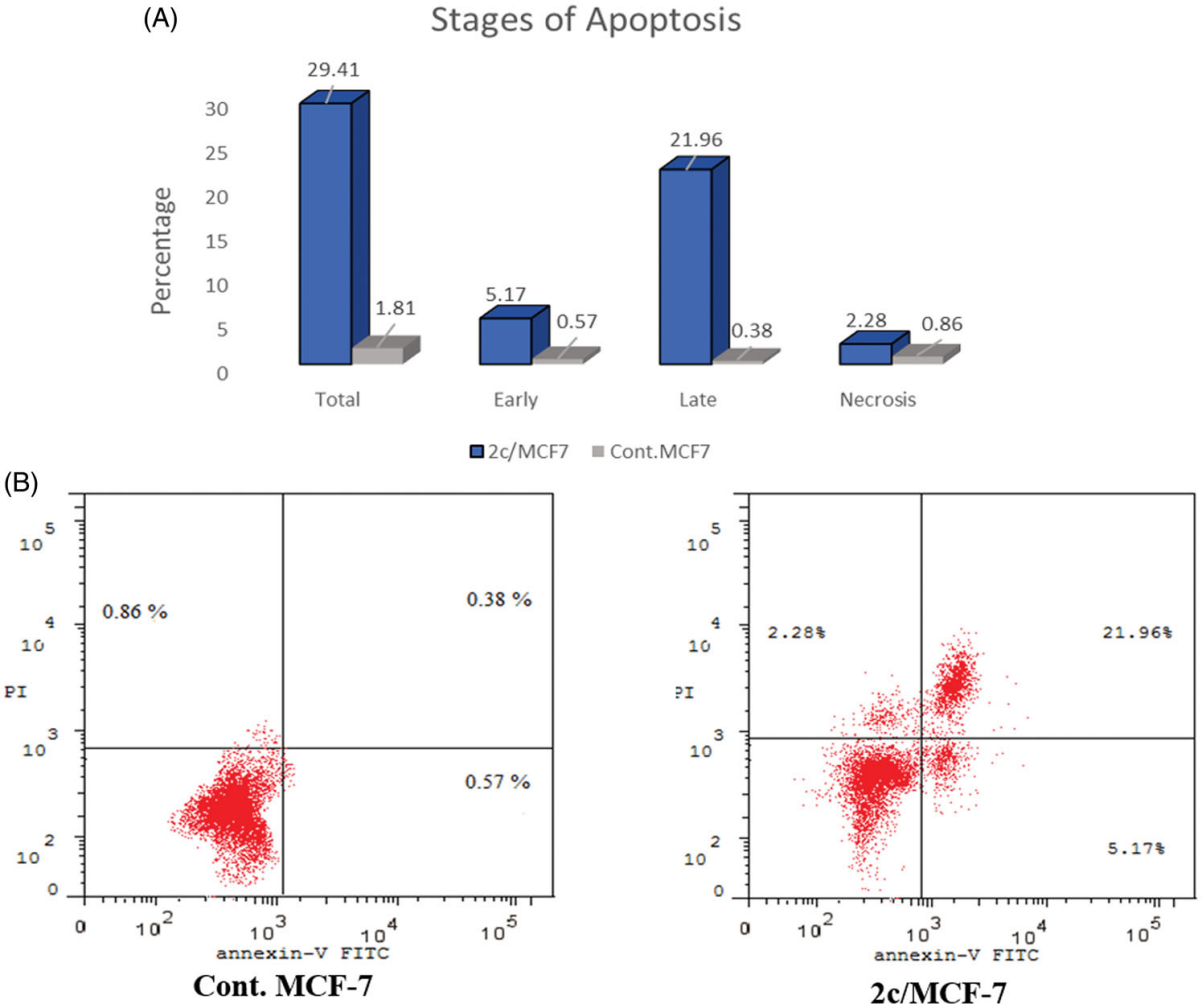
(A). Percentage and stages of induced apoptosis in control MFC-7 and MFC-7 treated with compound **2c**. (B) Representative dot plots of MCF-7 cells treated with **2c** (1.52 µM) for 24 h and analysed by flow cytometry after double staining of the cells with Annexin-V-FITC and PI.

Annexin V-FITC and Propidium iodide (PI) double-staining assay is a helpful method for determining whether cell death is due to programmed apoptosis or to uncontrolled necrosis. Dual staining for Annexin-V and PI permits discrimination between live cells, early apoptotic cells, late apoptotic cells and necrotic cells. Annexin V is a protein with high affinity for phosphatidylserine (PS). The latter is a cell membrane element that translocate from the inner face of the plasma membrane to the cell surface after initiating apoptosis. Once on the cell surface, PS can be detected by fluorescent Annexin V conjugate. On the other hand, PI stains DNA and penetrates only dead cells^58^. As shown in Figure 6, after 24 h of treatment of MCF-7 cells with compound **2c** at its IC_50_ concentration (1.52 µM) a decrease in the percentage of the survived cells was detected. The results showed an increase in the apoptotic cells percentage in early apoptosis phase from 0.57% to 5.17% (9.0-fold more than control) and a significant elevation in late apoptosis phase from 0.38% to 21.96% (57.78-fold more than control) which merge on the intrinsic and extrinsic pathways. Moreover, this corresponds to an increase in the percentage of total apoptosis from 1.81% to 29.41% (16.24-fold, compared to the control).

### Reactive oxygen species (ROS)

Reactive oxygen species (ROS) including superoxide anion (O_2_^−^), hydroxyl radicals (HO**^.^**), and hydrogen peroxide (H_2_O_2_) are produced inside the cells as byproducts of metabolic processes. Moderate levels of ROS are beneficial to the cell and are used in the defensing mechanisms against pathogenic infection as well as wound healing and repairing mechanisms^59^. Normal healthy cells employ antioxidant defense system to detoxify extra ROS to maintain the balance between oxidant and antioxidant species^60^. The oxidative stress occurs due to the accumulation of ROS in the cell when the production of ROS exceeds the ability of the cell to maintain the balance between its level and antioxidants. The accumulation of ROS can cause cell death due to its damaging effects on lipids, proteins and cellular DNA^61^. Moreover, the accumulation of ROS activates cellular pathways which affect cell proliferation, differentiation, and apoptosis including NF-_k_B and p38 MAPK pathways^62^. The anticancer activity of most chemotherapeutic agents is explained to be due to their ability to increase the level of ROS in cancer cells over a threshold to induce cell death^63^.

**Figure 7.**
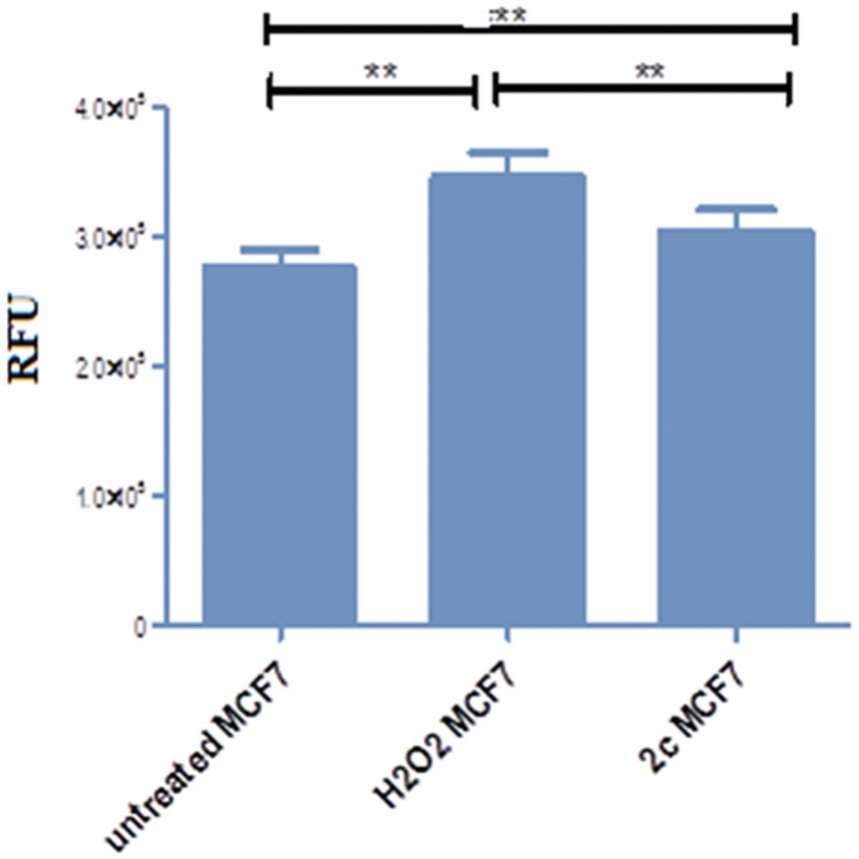
Graphical representation to show the increase of intercellular ROS in **2c** treated MCF-7 cells compared to H_2_O_2_ treated MCF-7 and untreated MCF-7 cells. ROS content was reported as relative fluorescence units (RFU).

The ability of many chalcone derivatives to act as pro-oxidant was documented in the literature^64^. This action is thought to be linked to their cytotoxic behaviour in various cell lines^65^. The induction of oxidative stress through production of ROS causes the damage of DNA and proteins. Production of phenoxide radicle of chalcone derivatives may constitute the main ROS agent^66^. In this investigation and as a result of the existence of phenoxy moiety in the synthesised chalcone derivatives, ROS production of compound **2c** was evaluated on MCF-7 cell line using 1.52 µM and the results were displayed in Figure 7. As a result of this evaluation, compound **2c** can induce ROS by 10% higher than control in MCF-7 cells.

### Evaluation of p38 MAPK

The p38 MAPK is one group of kinase family with a critical role in cancer progression^67^. Down-regulation of this enzyme was associated with diminished survival of breast cancer cells^68^. In a previous study, Parekh et al. observed that liver cancer cell development has high p38 and after using resveratrol both total and phosphorylated p38 were down-regulated^69^. Down-regulation of p38 MAPK regulates cell apoptosis and oxidative stress by expression of pro-inflammatory and G2/M checkpoint regulation leading to DNA damage which decrease cancer growth and invasiveness^70^.

**Figure 8.**
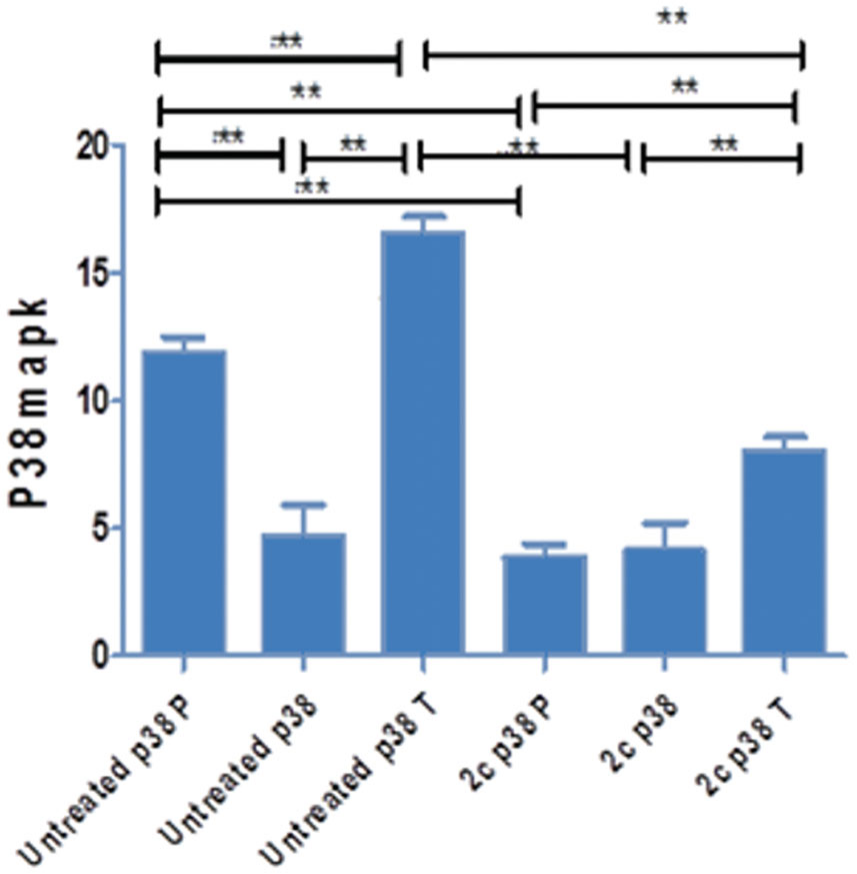
Graphical representation to show that **2c** treated MCF-7 cells decreased both total p38 MAPK (p38T) and phosphorylated p38 MAPK (p38P) compared to untreated MCF-7 cells.

The concentration of phosphorylated and total p38 MAPK was evaluated in MCF-7 cells using 1.52 µM of compound **2c** to assess its ability to down-regulate this key enzyme. The results indicated that compound **2c** can down-regulate total p38 MAPK by almost 40% and can interfere with its phosphorylation by almost 60% (Figure 8). Compound **2c** decreased both total p38 MAPK (p38T, Figure 8) and phosphorylated p38 MAPK (p38P, Figure 8) in MCF-7 treated cells when compared to untreated MCF-7 cells which suggests that compound **2c** caused inhibition of cell proliferation, growth, and survival. Down-regulation of p38 MAPK as well as induction of ROS production may explain the cytotoxic activity of compound **2c**.

### Molecular docking study

**Figure 9.**
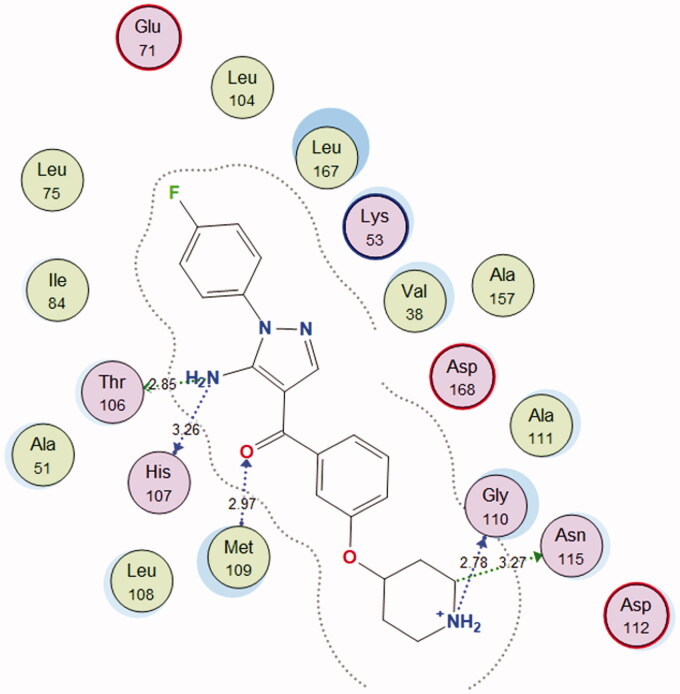
2D interactions of PQA within p38alpha MAP kinase active site.

In the search for the possible mechanism of the compounds in inhibition of p38 MAPK pathway, chalcones **2c** and **2f** that showed the best IC_50_ values against MCF-7 cell line were docked to the crystal structure of p38alpha MAP kinase complexed with [5-amino-1–(4-fluorophenyl)-1H-pyrazol-4-yl][3-(piperidin-4-yloxy)phenyl]methanone (PQA) ligand (PDB code: 2BAL)^42^ using MOE, 2019.0102 software. Through examination of the binding interactions of PQA to the active site of the enzyme, it shows strong hydrogen bond interactions with Thr106, His107, Met109 & Gly110 (Figure 9).

**Figure 10.**
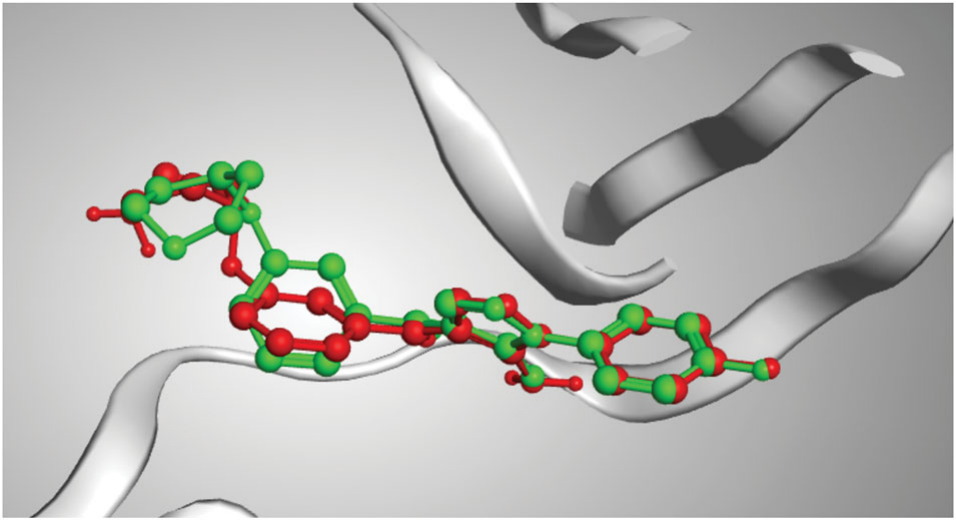
3D representation of the superimposition of the co-crystallised (red) and the docking pose (green) of PQA in the active site of p38alpha MAP kinase enzyme.

Docking setup was first validated by self-docking of the co-crystallised ligand (PQA) in the vicinity of the binding site of the enzyme, the docking score (S) was −11.9710 kcal/mol. and root mean square deviation (RMSD) was 1.7685 Å (Figure 10).

**Figure 11.**
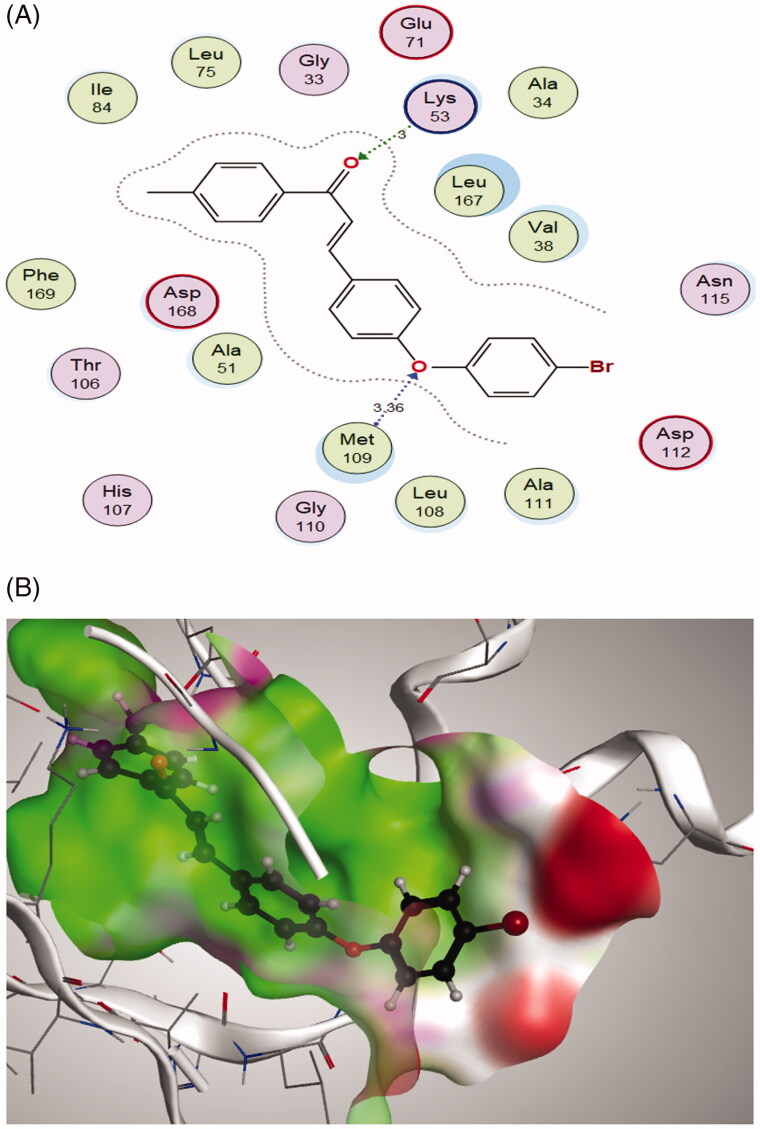
(A) 2 D interactions of compound **2c** within p38alpha MAP kinase active site; (B) 3 D diagram of compound **2c** interactions within p38alpha MAP kinase active site.

**Figure 12.**
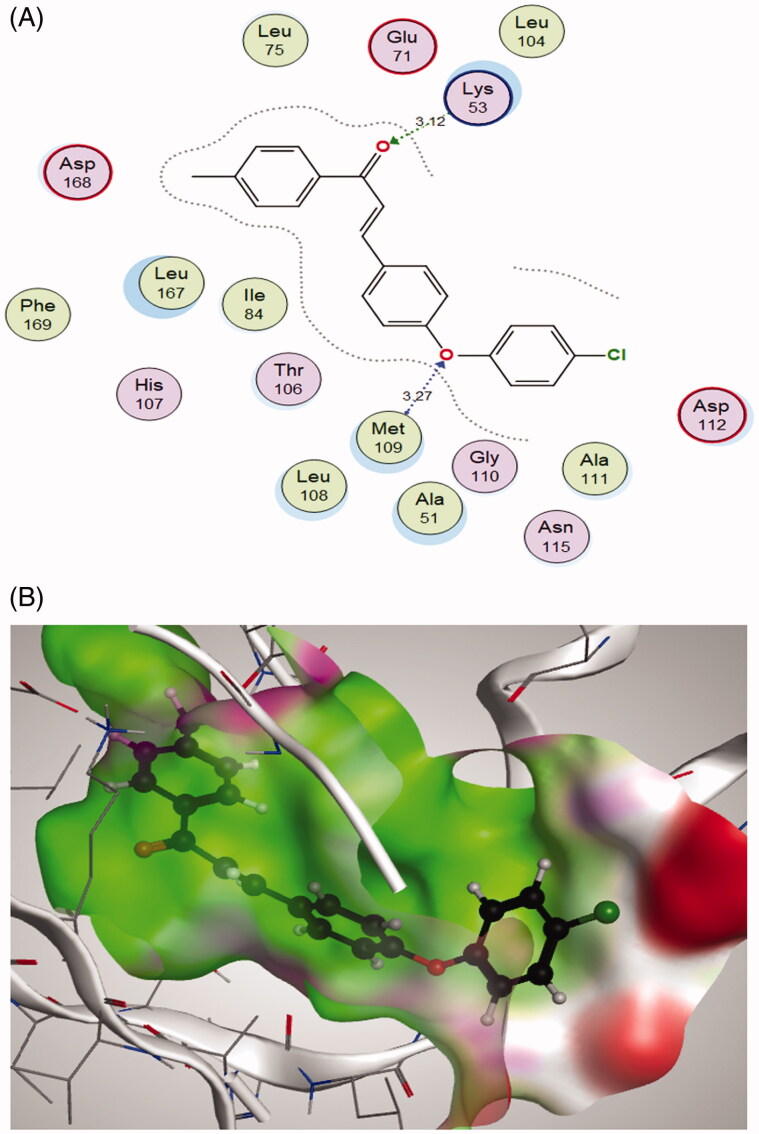
(A) 2 D interactions of compound **2f** within p38alpha MAP kinase active site; (B) 3 D diagram of compound **2f** interactions within p38alpha MAP kinase active site.

Both tested compounds **2c** and **2f** showed good binding energy score which is similar to the bound ligand (−11.5298 and −11.3114 kcal/mol., respectively). They showed binding interactions with Lys53 and the key amino acid Met109. Moreover, they showed good fitting in the enzyme pocket which is clarified in the 3 D pictures of the best docking poses. The results are summarised in Table 2 and Figures 11 and 12).

**Table 2.** Docking scores, hydrogen bonds and interactions of tested compounds **2c** and **2f** inside p38alpha MAP kinase binding site (PDB entry 2BAL).

Compound	S (kcal/mol)	Amino acids	Interacting groups	Type of interaction	Length
**2c**	−11.5298	Lys53	O (C=O)	H-bond acceptor	3.00
		Met109	O (Ether)	H-bond acceptor	3.36
**2f**	−11.3114	Lys53	O (C=O)	H-bond acceptor	3.12
		Met109	O (Ether)	H-bond acceptor	3.27

## Conclusion

A series of halogenated phenoxychalcones **2a–f** and their corresponding *N*-acetylpyrazolines **3a–f** was synthesised and evaluated for their anticancer activity against MCF-7 breast cancer cell line. The synthesised compounds showed significant cytotoxic activity and the most promising was compound **2c** with IC_50_ of 1.52 µM and 15-fold selectivity towards breast cancer cell line over normal breast cell line. Compound **2c** down-regulated p38α MAPK and induced ROS in MCF-7 cell line. Direct interaction of compound **2c** with p38alpha MAP Kinase active sites was revealed by molecular docking studies. It also caused cell cycle arrest in G2/M phase which indicates its apoptotic behaviour. The same compound induced significant increase in apoptotic cells in late apoptosis phase by 57.78-fold when compared to control. The investigation of chemical modifications of the chalcone structure by introducing electron withdrawing, meta directing groups such as nitro group in different positions would be of great value. Furthermore, the evaluation of the anti-tubulin polymerisation of the synthesised diaryl ether chalcones would be of great importance for better understanding of the biological activity.

## Supplementary Material

Supplemental Material
