# Supplementary material for: Synthesis and biological evaluation of halogenated phenoxychalcones and their corresponding pyrazolines as cytotoxic agents in human breast cancer
Source: J Enzyme Inhib Med Chem. 2021 Dec 11;37(1):189–201. doi: 10.1080/14756366.2021.1998023 (PMC8667918; doi:10.1080/14756366.2021.1998023)
Supplement: Supplemental Material [file IENZ_A_1998023_SM6286.pdf]

Compound 2a

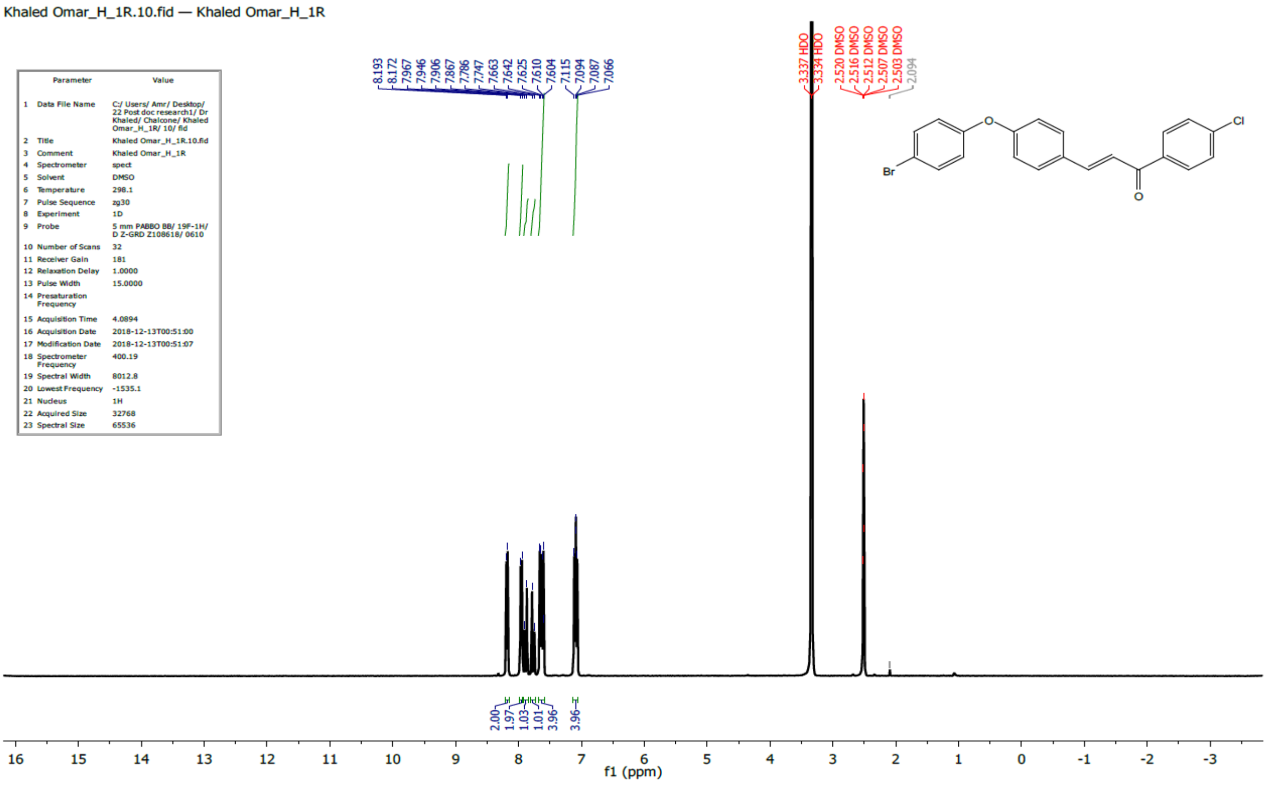
Khaled Omar_H_1R.10.fid — Khaled Omar_H_1R
8.193
8.172
7.967
7.946
7.906
7.867
7.867
7.786
7.747
7.663
7.642
7.625
7.610
7.604
7.115
7.094
7.087
7.066
3.337 HDO
3.334 HDO
2.520 DMSO
2.516 DMSO
2.512 DMSO
2.507 DMSO
2.503 DMSO
2.094
Br
O
Cl
O
2.00
1.97
1.03
1.01
3.96
3.96
16 15 14 13 12 11 10 9 8 7 6 5 4 3 2 1 0 -1 -2 -3
f1 (ppm)
Parameter | Value
1 Data File Name | C:/ Users/ Amr/ Desktop/ 22 Post doc research1/ Dr Khaled/ Chalcone/ Khaled Omar_H_1R/ 10/ fid
2 Title | Khaled Omar_H_1R.10.fid
3 Comment | Khaled Omar_H_1R
4 Spectrometer | spect
5 Solvent | DMSO
6 Temperature | 298.1
7 Pulse Sequence | zg30
8 Experiment | 1D
9 Probe | 5 mm PABBO BB/ 19F-1H/ D Z-GRD Z108618/ 0610
10 Number of Scans | 32
11 Receiver Gain | 181
12 Relaxation Delay | 1.0000
13 Pulse Width | 15.0000
14 Presaturation Frequency |
15 Acquisition Time | 4.0894
16 Acquisition Date | 2018-12-13T00:51:00
17 Modification Date | 2018-12-13T00:51:07
18 Spectrometer Frequency | 400.19
19 Spectral Width | 8012.8
20 Lowest Frequency | -1535.1
21 Nucleus | 1H
22 Acquired Size | 32768
23 Spectral Size | 65536

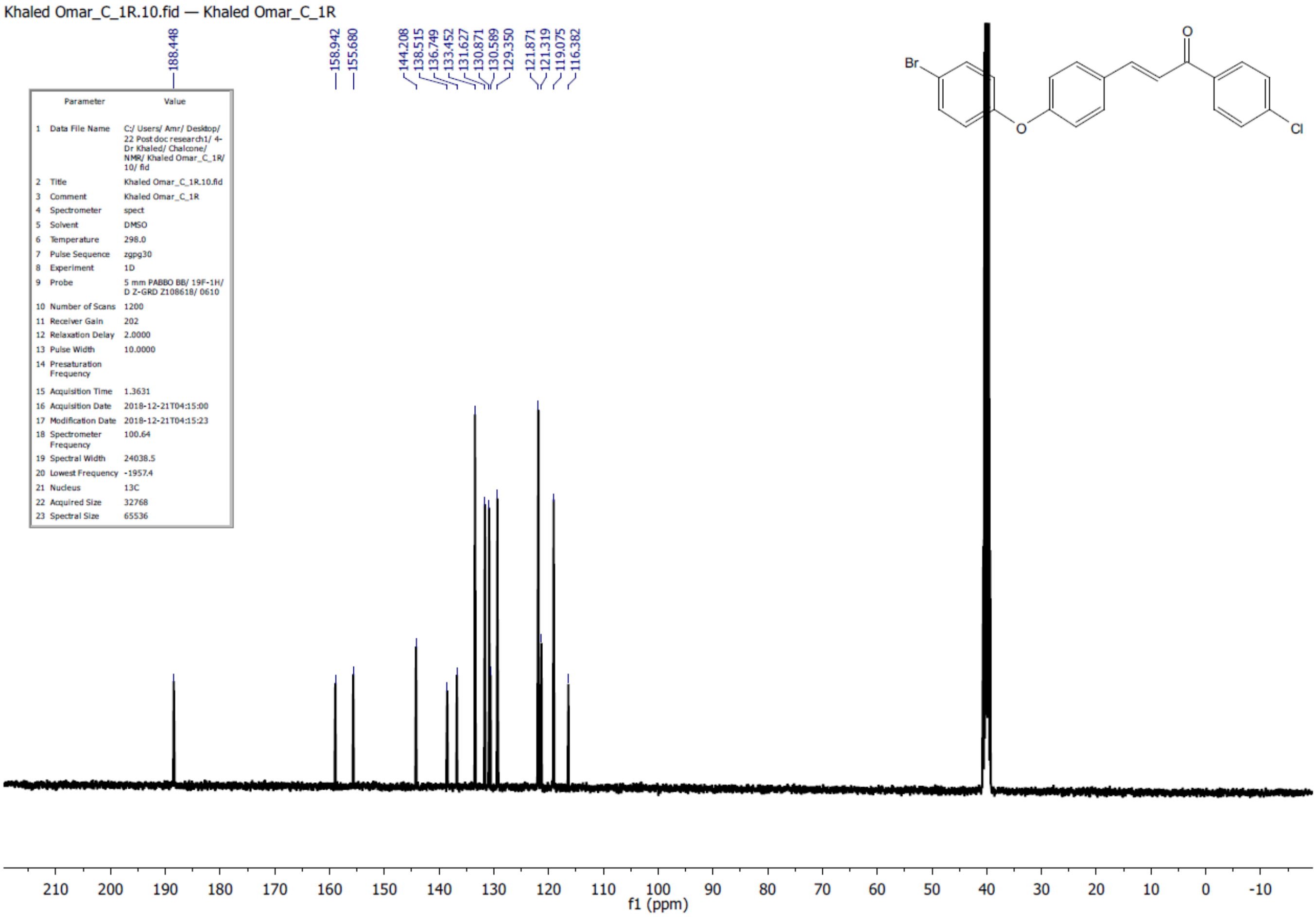
Khaled Omar_C_1R.10.fid — Khaled Omar_C_1R
188.448
158.942
155.680
144.208
138.515
136.749
133.452
131.627
130.871
130.589
129.350
121.871
121.319
119.075
116.382
Br
O
O
Cl
210 200 190 180 170 160 150 140 130 120 110 100 90 80 70 60 50 40 30 20 10 0 -10
f1 (ppm)
Parameter | Value
1 Data File Name | C:/ Users/ Amr/ Desktop/ 22 Post doc research1/ 4-Dr Khaled/ Chalcone/ NMR/ Khaled Omar_C_1R/ 10/ fid
2 Title | Khaled Omar_C_1R.10.fid
3 Comment | Khaled Omar_C_1R
4 Spectrometer | spect
5 Solvent | DMSO
6 Temperature | 298.0
7 Pulse Sequence | zgpg30
8 Experiment | 1D
9 Probe | 5 mm PABBO BB/ 19F-1H/ D Z-GRD Z108618/ 0610
10 Number of Scans | 1200
11 Receiver Gain | 202
12 Relaxation Delay | 2.0000
13 Pulse Width | 10.0000
14 Presaturation Frequency |
15 Acquisition Time | 1.3631
16 Acquisition Date | 2018-12-21T04:15:00
17 Modification Date | 2018-12-21T04:15:23
18 Spectrometer Frequency | 100.64
19 Spectral Width | 24038.5
20 Lowest Frequency | -1957.4
21 Nucleus | 13C
22 Acquired Size | 32768
23 Spectral Size | 65536

Compound 2b

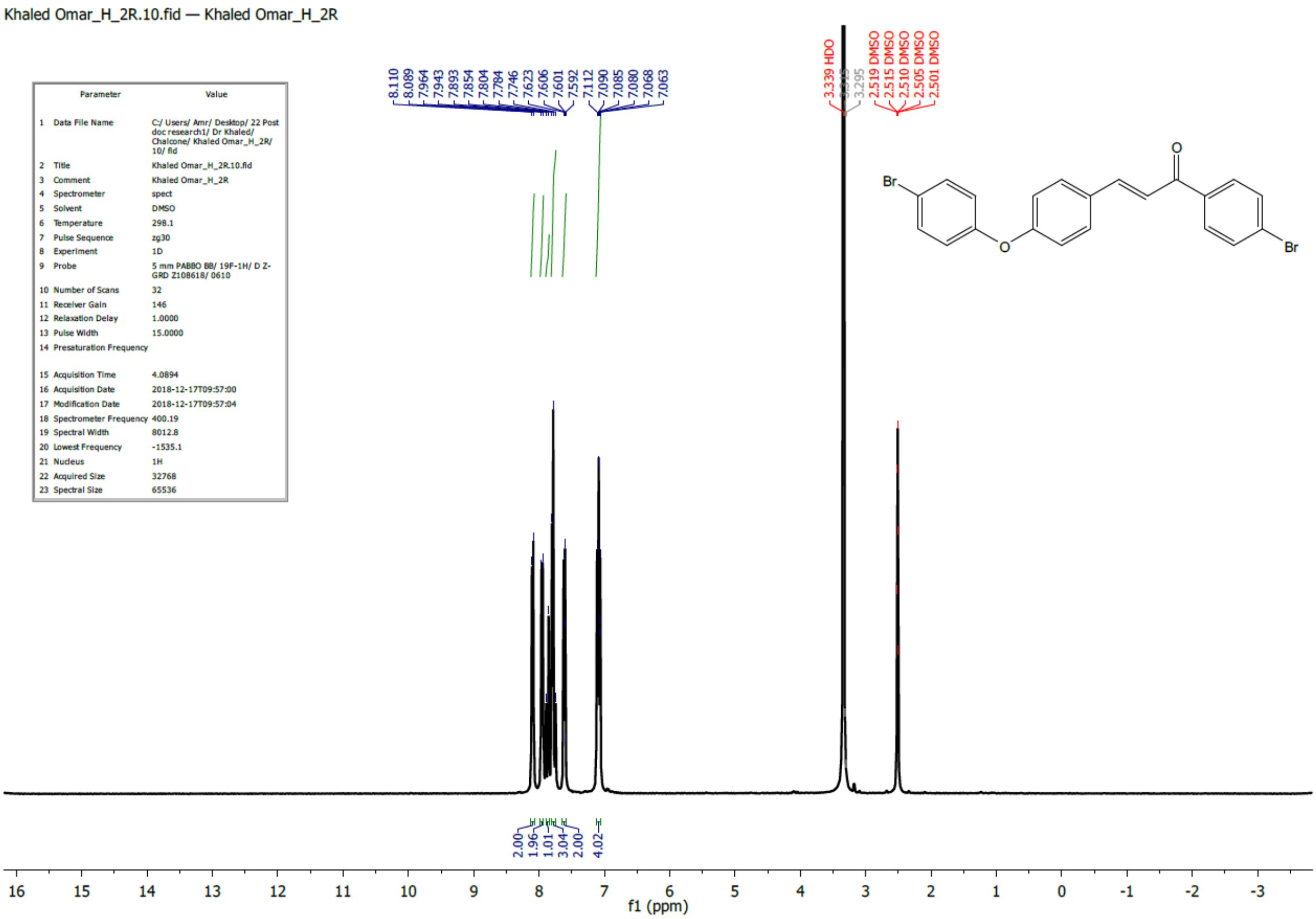
Khaled Omar_H_2R.10.fid — Khaled Omar_H_2R
Br
O
O
Br
f1 (ppm)

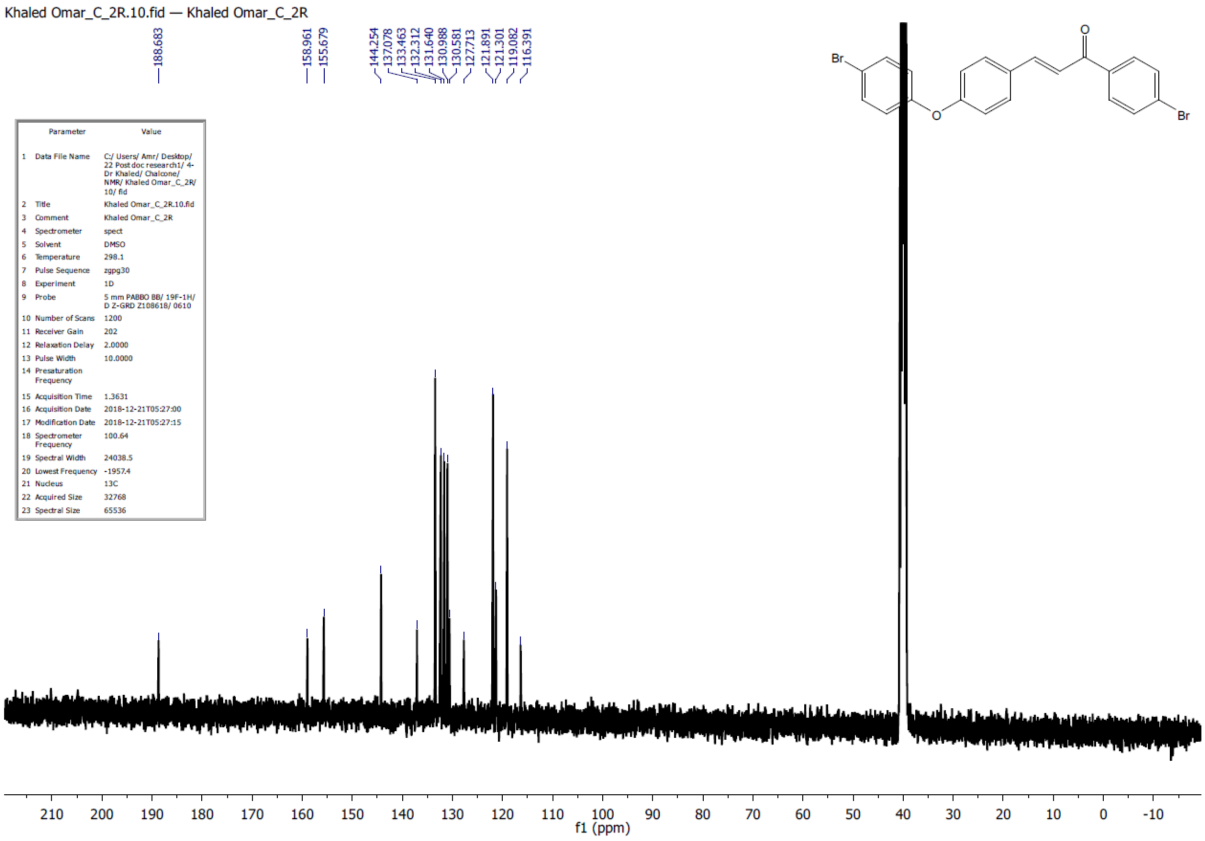
Khaled Omar_C_2R.10.fid — Khaled Omar_C_2R
188.683
158.961
155.679
144.254
137.078
133.463
132.312
131.640
130.988
130.581
127.713
121.891
121.301
119.082
116.391
Br
O
O
Br
f1 (ppm)

Compound 2c

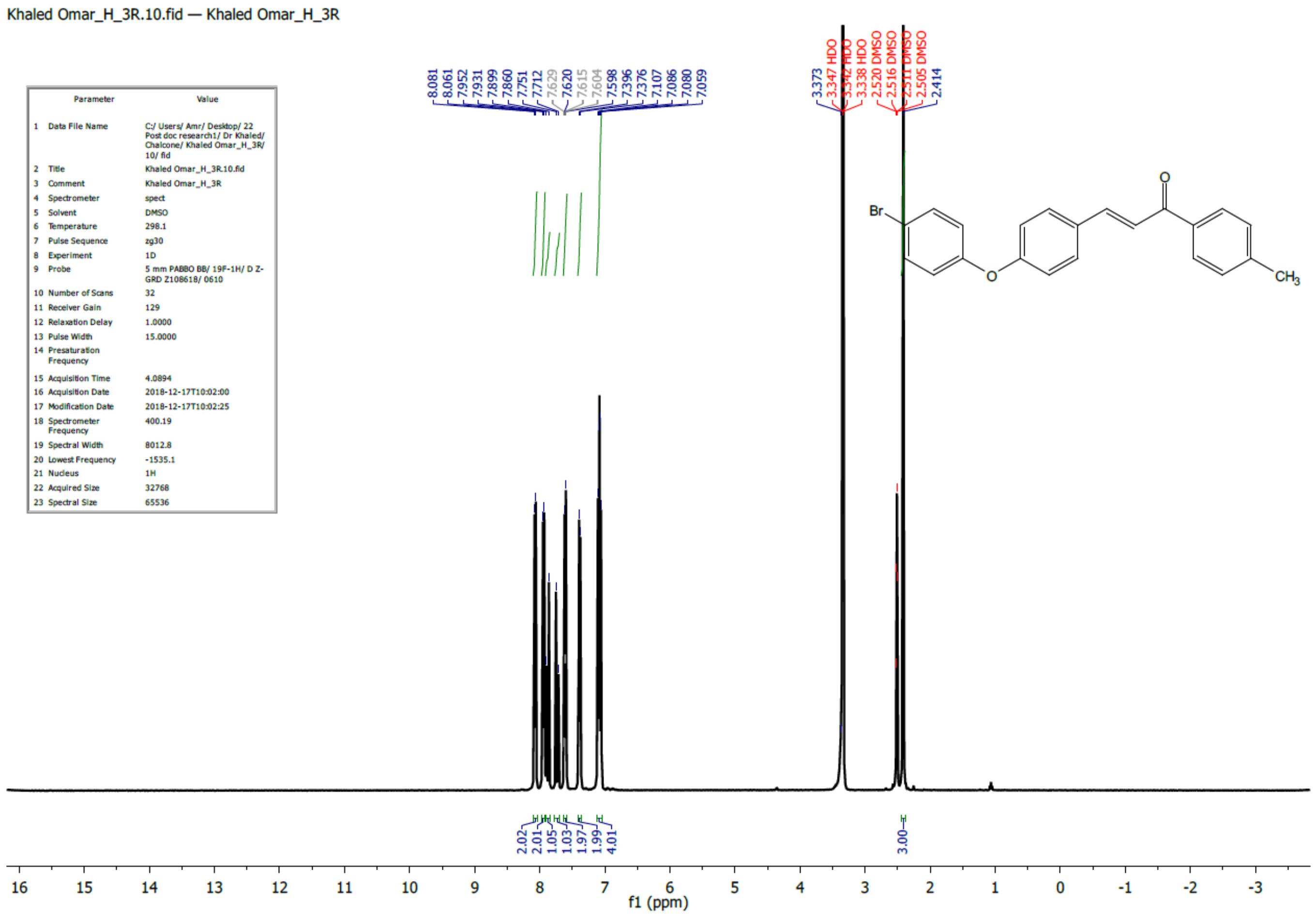

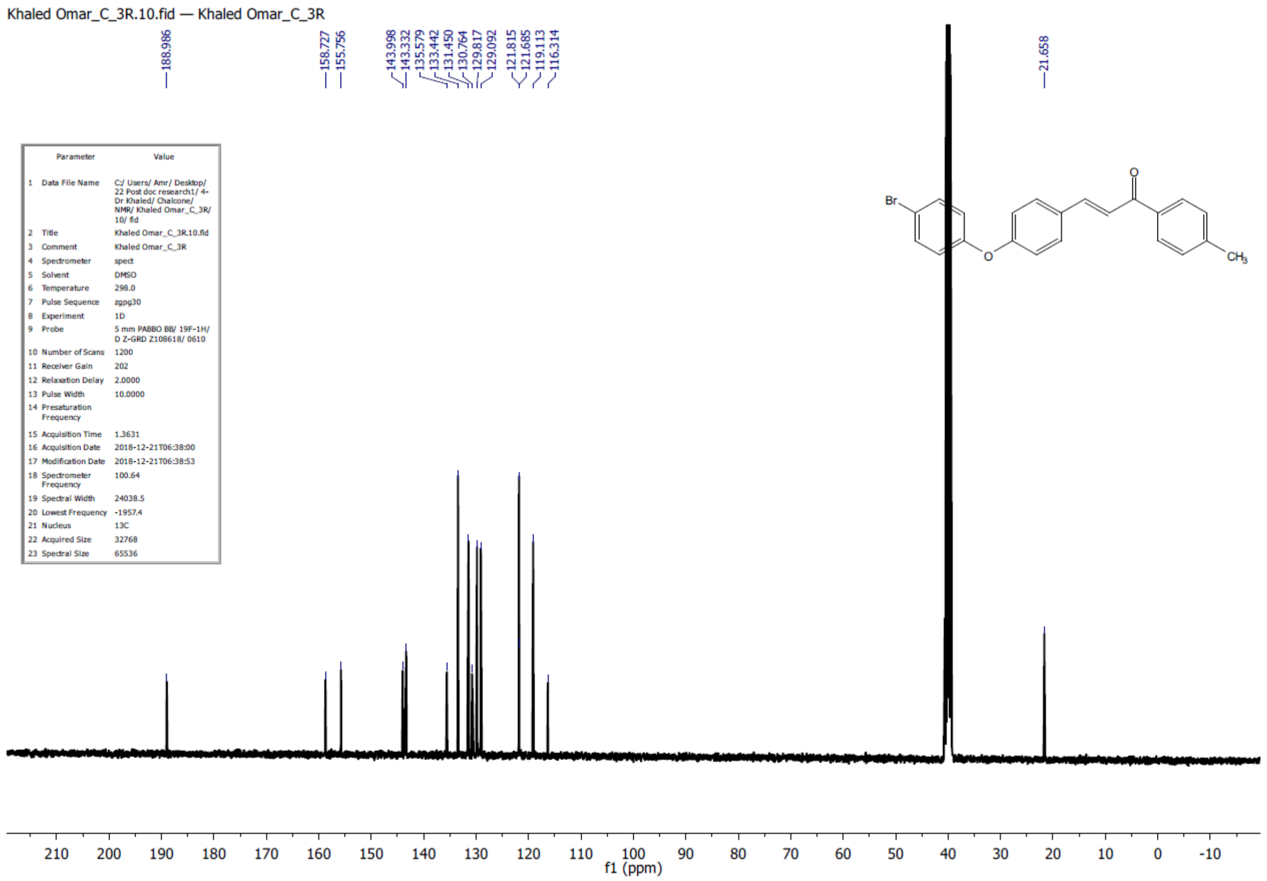

Compound 2d

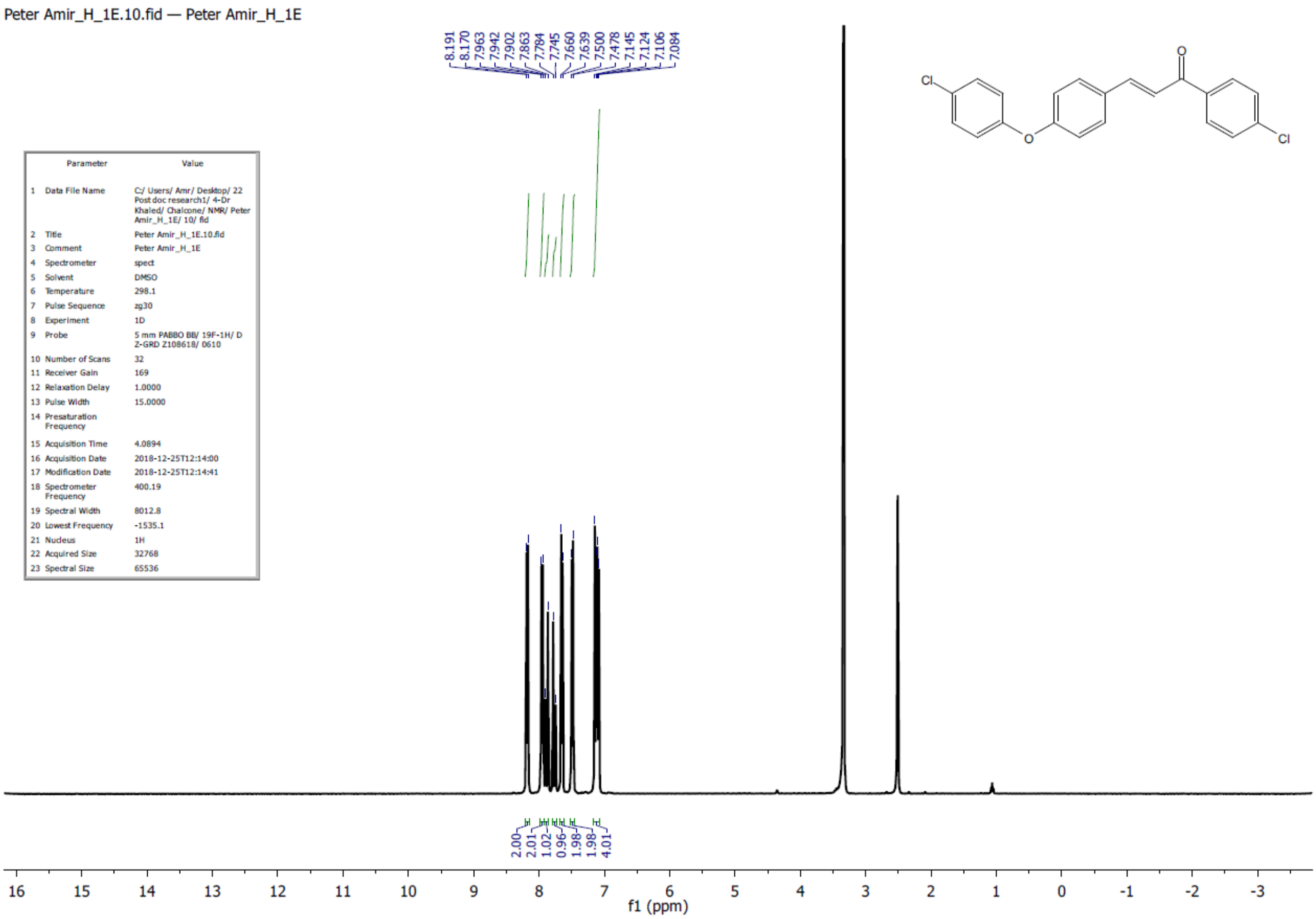

Peter Amir_H_1E.10.fid — Peter Amir_H_1E
8.191
8.170
7.963
7.942
7.902
7.863
7.784
7.745
7.660
7.639
7.500
7.478
7.145
7.124
7.106
7.084
Cl
O
Cl
Parameter | Value
1 Data File Name | C:/ Users/ Amr/ Desktop/ 22 Post doc research1/ 4-Dr Khaled/ Chalcone/ NMR/ Peter Amir_H_1E/ 10/ fid
2 Title | Peter Amir_H_1E.10.fid
3 Comment | Peter Amir_H_1E
4 Spectrometer | spect
5 Solvent | DMSO
6 Temperature | 298.1
7 Pulse Sequence | zg30
8 Experiment | 1D
9 Probe | 5 mm PABBO BB/ 19F-1H/ D Z-GRD Z108618/ 0610
10 Number of Scans | 32
11 Receiver Gain | 169
12 Relaxation Delay | 1.0000
13 Pulse Width | 15.0000
14 Presaturation Frequency |
15 Acquisition Time | 4.0894
16 Acquisition Date | 2018-12-25T12:14:00
17 Modification Date | 2018-12-25T12:14:41
18 Spectrometer Frequency | 400.19
19 Spectral Width | 8012.8
20 Lowest Frequency | -1535.1
21 Nucleus | 1H
22 Acquired Size | 32768
23 Spectral Size | 65536
2.00
2.01
1.02
0.96
1.98
1.98
4.01
16 15 14 13 12 11 10 9 8 7 6 5 4 3 2 1 0 -1 -2 -3
f1 (ppm)

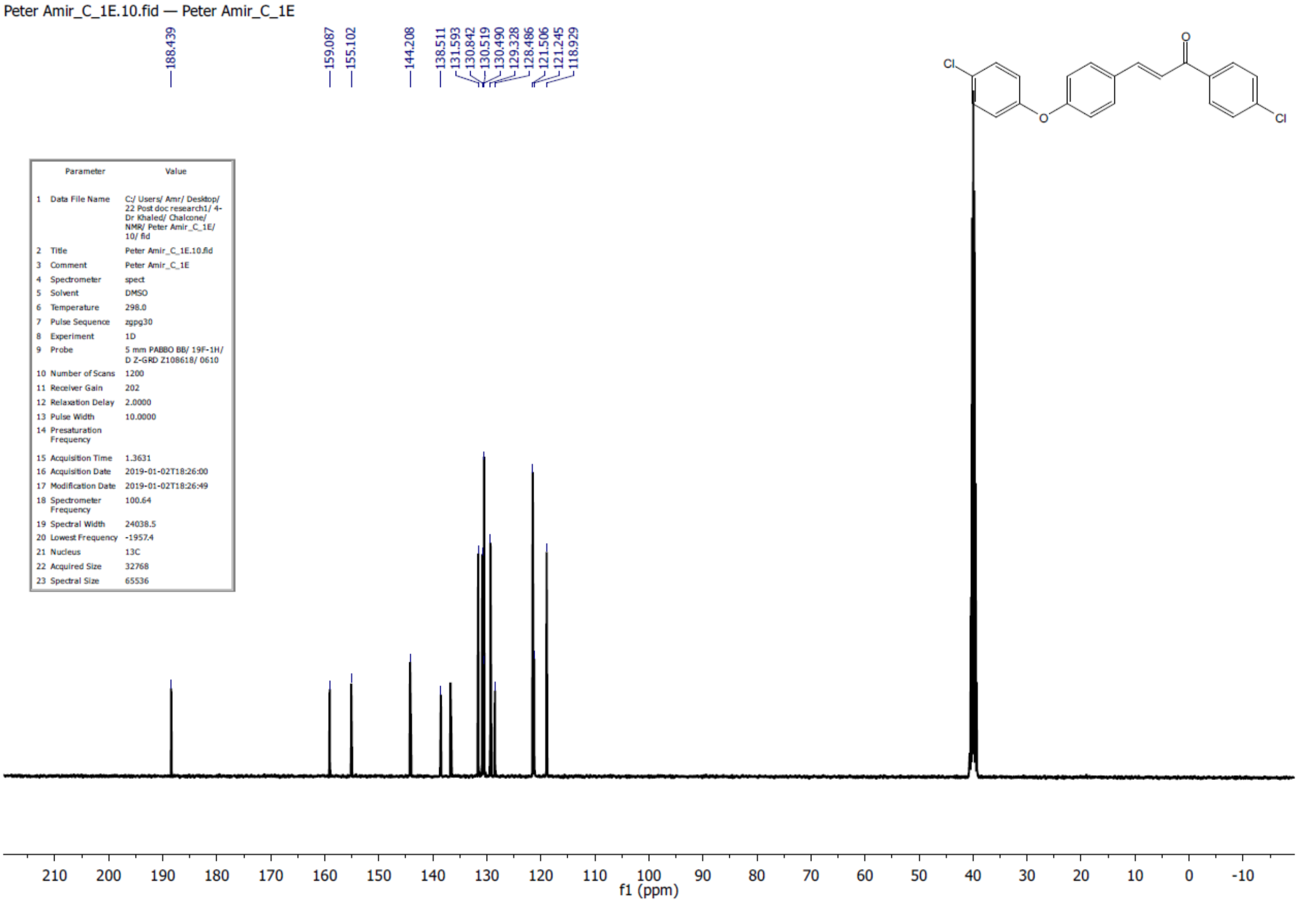

Peter Amir_C_1E.10.fid — Peter Amir_C_1E
188.439
159.087
155.102
144.208
138.511
131.593
130.842
130.519
130.490
129.328
128.486
121.506
121.245
118.929
Cl
O
Cl
Parameter | Value
1 Data File Name | C:/ Users/ Amr/ Desktop/ 22 Post doc research1/ 4-Dr Khaled/ Chalcone/ NMR/ Peter Amir_C_1E/ 10/ fid
2 Title | Peter Amir_C_1E.10.fid
3 Comment | Peter Amir_C_1E
4 Spectrometer | spect
5 Solvent | DMSO
6 Temperature | 298.0
7 Pulse Sequence | zgpg30
8 Experiment | 1D
9 Probe | 5 mm PABBO BB/ 19F-1H/ D Z-GRD Z108618/ 0610
10 Number of Scans | 1200
11 Receiver Gain | 202
12 Relaxation Delay | 2.0000
13 Pulse Width | 10.0000
14 Presaturation Frequency |
15 Acquisition Time | 1.3631
16 Acquisition Date | 2019-01-02T18:26:00
17 Modification Date | 2019-01-02T18:26:49
18 Spectrometer Frequency | 100.64
19 Spectral Width | 24038.5
20 Lowest Frequency | -1957.4
21 Nucleus | 13C
22 Acquired Size | 32768
23 Spectral Size | 65536
210 200 190 180 170 160 150 140 130 120 110 100 90 80 70 60 50 40 30 20 10 0 -10
f1 (ppm)

Compound 2e

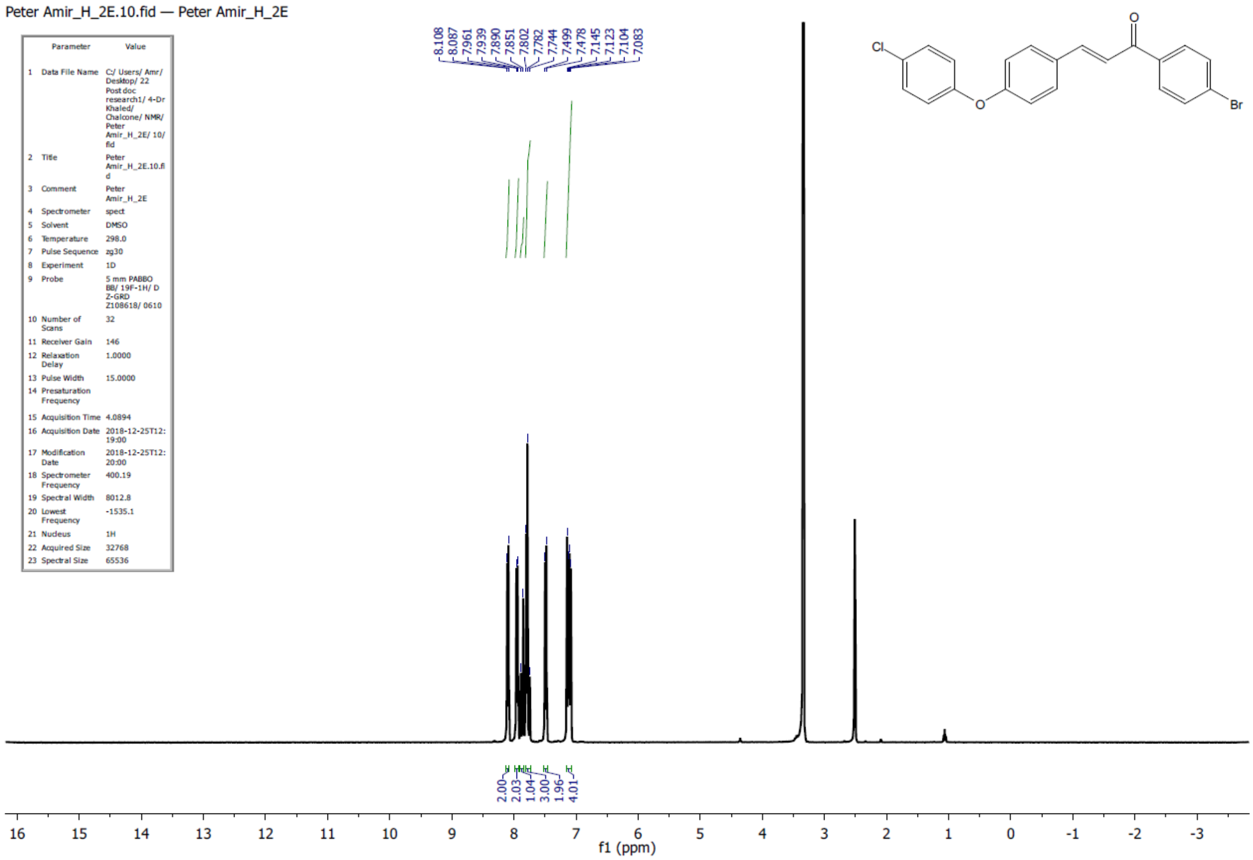

Peter Amir_H_2E.10.fid — Peter Amir_H_2E
8.108
8.087
7.961
7.939
7.890
7.851
7.802
7.782
7.744
7.499
7.478
7.145
7.123
7.104
7.083
Cl
O
O
Br
Parameter Value
1 Data File Name C:/ Users/ Amr/ Desktop/ 22 Post doc research1/ 4-Dr Khaled/ Chalcone/ NMR/ Peter Amir_H_2E/ 10/ fid
2 Title Peter Amir_H_2E.10.fid
3 Comment Peter Amir_H_2E
4 Spectrometer spect
5 Solvent DMSO
6 Temperature 298.0
7 Pulse Sequence zg30
8 Experiment 1D
9 Probe 5 mm PABBO BB/ 19F-1H/ D Z-GRD Z108618/ 0610
10 Number of Scans 32
11 Receiver Gain 146
12 Relaxation Delay 1.0000
13 Pulse Width 15.0000
14 Presaturation Frequency
15 Acquisition Time 4.0894
16 Acquisition Date 2018-12-25T12:19:00
17 Modification Date 2018-12-25T12:20:00
18 Spectrometer Frequency 400.19
19 Spectral Width 8012.8
20 Lowest Frequency -1535.1
21 Nucleus 1H
22 Acquired Size 32768
23 Spectral Size 65536
2.00
2.03
1.04
3.00
1.96
4.01
16 15 14 13 12 11 10 9 8 7 6 5 4 3 2 1 0 -1 -2 -3
f1 (ppm)

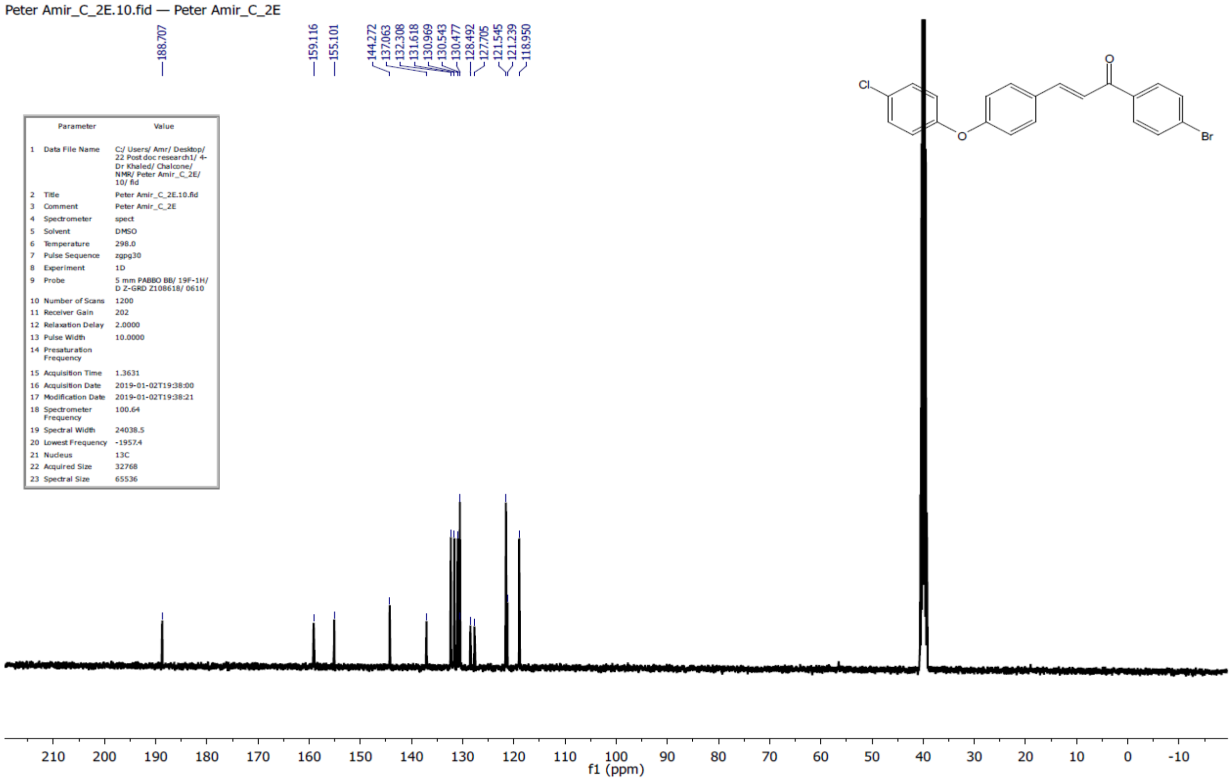

Peter Amir_C_2E.10.fid — Peter Amir_C_2E
188.707
159.116
155.101
144.272
137.063
132.308
131.618
130.969
130.543
130.477
128.492
127.705
121.545
121.239
118.950
Cl
O
O
Br
Parameter Value
1 Data File Name C:/ Users/ Amr/ Desktop/ 22 Post doc research1/ 4-Dr Khaled/ Chalcone/ NMR/ Peter Amir_C_2E/ 10/ fid
2 Title Peter Amir_C_2E.10.fid
3 Comment Peter Amir_C_2E
4 Spectrometer spect
5 Solvent DMSO
6 Temperature 298.0
7 Pulse Sequence zgpg30
8 Experiment 1D
9 Probe 5 mm PABBO BB/ 19F-1H/ D Z-GRD Z108618/ 0610
10 Number of Scans 1200
11 Receiver Gain 202
12 Relaxation Delay 2.0000
13 Pulse Width 10.0000
14 Presaturation Frequency
15 Acquisition Time 1.3631
16 Acquisition Date 2019-01-02T19:38:00
17 Modification Date 2019-01-02T19:38:21
18 Spectrometer Frequency 100.64
19 Spectral Width 24038.5
20 Lowest Frequency -1957.4
21 Nucleus 13C
22 Acquired Size 32768
23 Spectral Size 65536
210 200 190 180 170 160 150 140 130 120 110 100 90 80 70 60 50 40 30 20 10 0 -10
f1 (ppm)

Compound 2f

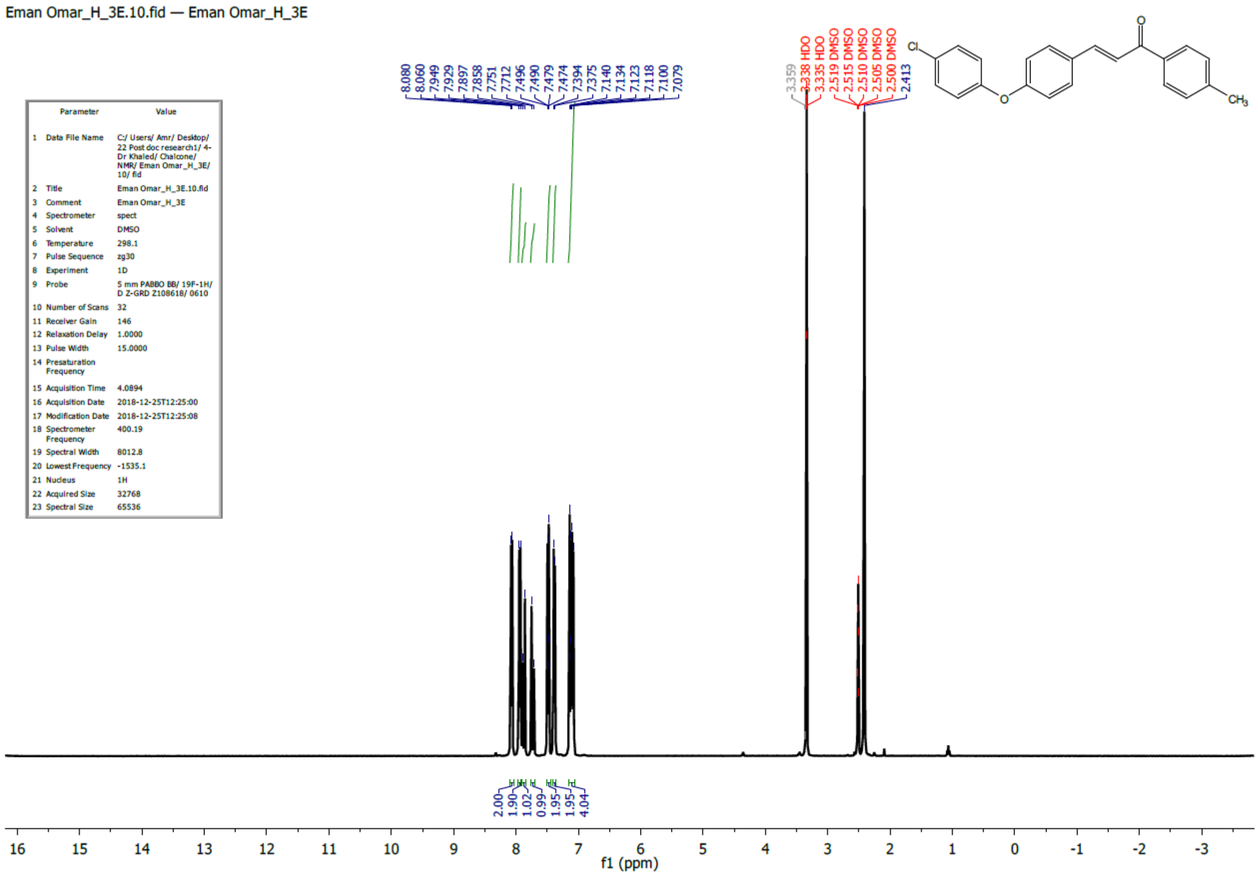
Eman Omar_H_3E.10.fid — Eman Omar_H_3E
Parameter Value
1 Data File Name C:/ Users/ Amr/ Desktop/ 22 Post doc research1/ 4-Dr Khaled/ Chalcone/ NMR/ Eman Omar_H_3E/ 10/ fid
2 Title Eman Omar_H_3E.10.fid
3 Comment Eman Omar_H_3E
4 Spectrometer spect
5 Solvent DMSO
6 Temperature 298.1
7 Pulse Sequence zg30
8 Experiment 1D
9 Probe 5 mm PABBO BB/ 19F-1H/ D Z-GRD Z108618/ 0610
10 Number of Scans 32
11 Receiver Gain 146
12 Relaxation Delay 1.0000
13 Pulse Width 15.0000
14 Presaturation Frequency
15 Acquisition Time 4.0894
16 Acquisition Date 2018-12-25T12:25:00
17 Modification Date 2018-12-25T12:25:08
18 Spectrometer Frequency 400.19
19 Spectral Width 8012.8
20 Lowest Frequency -1535.1
21 Nucleus 1H
22 Acquired Size 32768
23 Spectral Size 65536
8.080 8.060 7.949 7.929 7.897 7.858 7.751 7.712 7.496 7.490 7.479 7.474 7.394 7.375 7.140 7.134 7.123 7.118 7.100 7.079
3.359 3.338 HDO 3.335 HDO 2.519 DMSO 2.515 DMSO 2.510 DMSO 2.505 DMSO 2.500 DMSO 2.413
Cl O O CH3
2.00 1.90 1.02 0.99 1.95 1.95 4.04
16 15 14 13 12 11 10 9 8 7 6 5 4 3 2 1 0 -1 -2 -3
f1 (ppm)

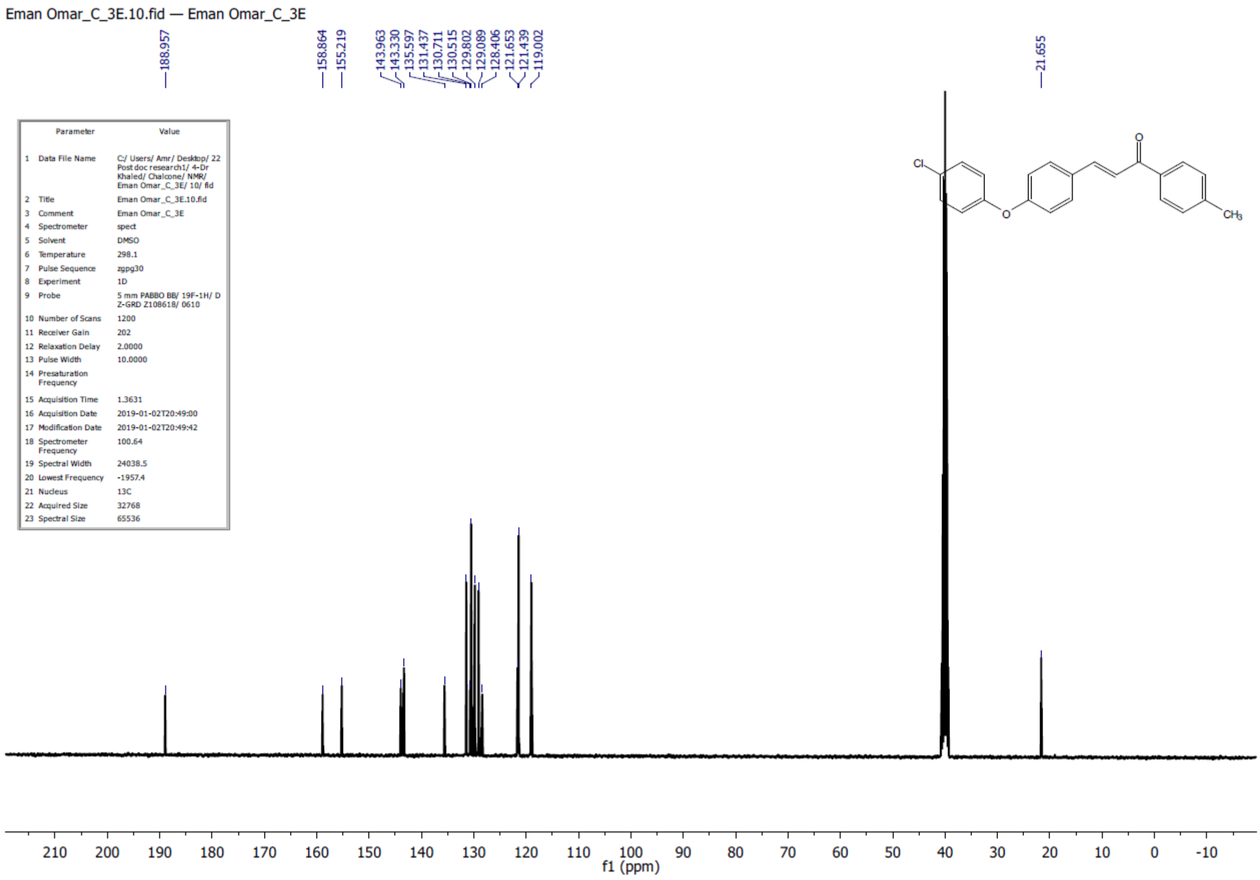
Eman Omar_C_3E.10.fid — Eman Omar_C_3E
188.957
158.864 155.219
143.963 143.330 135.597 131.437 130.711 130.515 129.802 129.089 128.406 121.653 121.439 119.002
21.655
Parameter Value
1 Data File Name C:/ Users/ Amr/ Desktop/ 22 Post doc research1/ 4-Dr Khaled/ Chalcone/ NMR/ Eman Omar_C_3E/ 10/ fid
2 Title Eman Omar_C_3E.10.fid
3 Comment Eman Omar_C_3E
4 Spectrometer spect
5 Solvent DMSO
6 Temperature 298.1
7 Pulse Sequence zgpg30
8 Experiment 1D
9 Probe 5 mm PABBO BB/ 19F-1H/ D Z-GRD Z108618/ 0610
10 Number of Scans 1200
11 Receiver Gain 202
12 Relaxation Delay 2.0000
13 Pulse Width 10.0000
14 Presaturation Frequency
15 Acquisition Time 1.3631
16 Acquisition Date 2019-01-02T20:49:00
17 Modification Date 2019-01-02T20:49:42
18 Spectrometer Frequency 100.64
19 Spectral Width 24038.5
20 Lowest Frequency -1957.4
21 Nucleus 13C
22 Acquired Size 32768
23 Spectral Size 65536
Cl O O CH3
210 200 190 180 170 160 150 140 130 120 110 100 90 80 70 60 50 40 30 20 10 0 -10
f1 (ppm)

Compound 3a

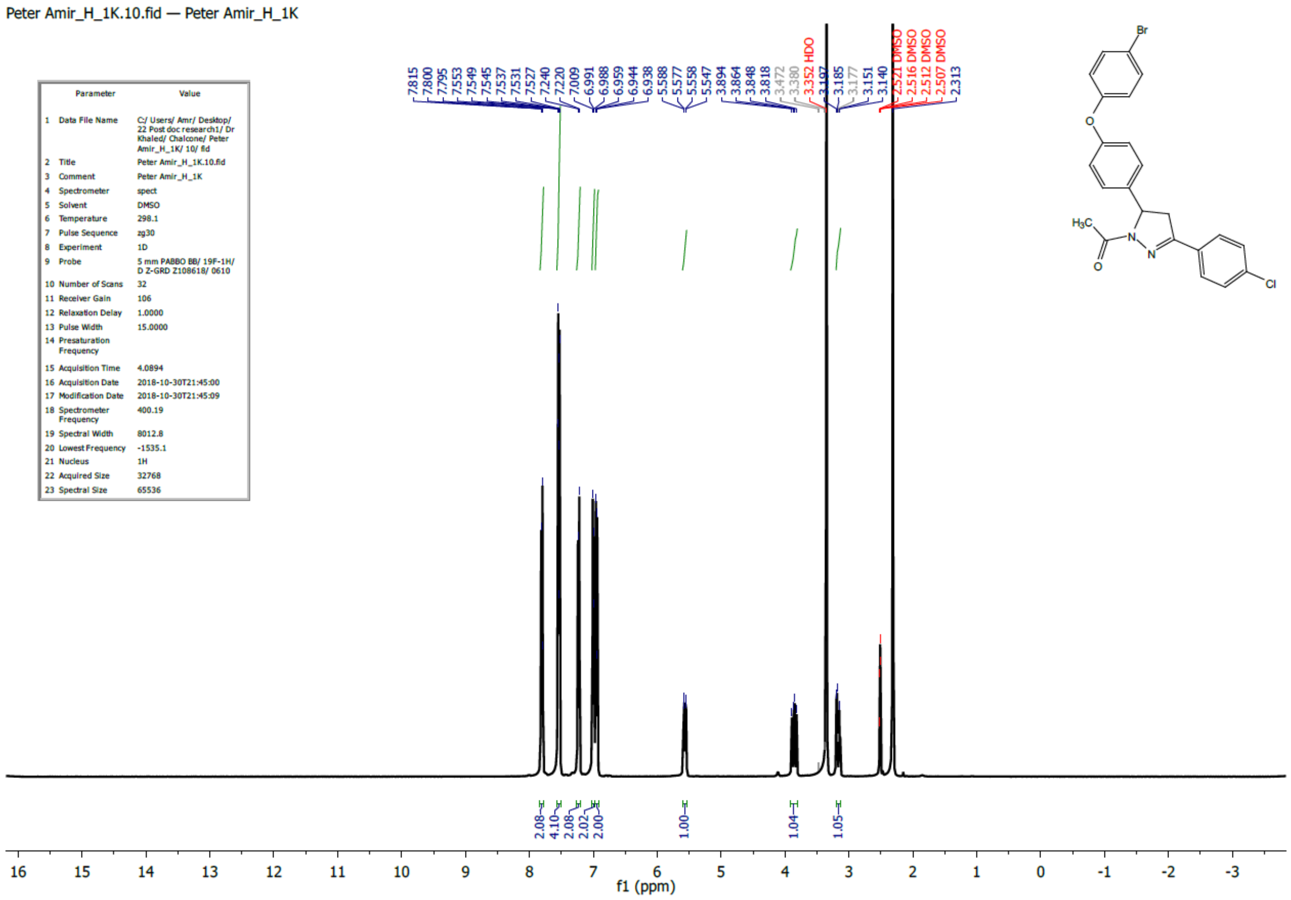
Peter Amir_H_1K.10.fid — Peter Amir_H_1K
Parameter Value
1 Data File Name C:/ Users/ Amr/ Desktop/ 22 Post doc research1/ Dr Khaled/ Chalcone/ Peter Amir_H_1K/ 10/ fid
2 Title Peter Amir_H_1K.10.fid
3 Comment Peter Amir_H_1K
4 Spectrometer spect
5 Solvent DMSO
6 Temperature 298.1
7 Pulse Sequence zg30
8 Experiment 1D
9 Probe 5 mm PABBO BB/ 19F-1H/ D Z-GRD Z108618/ 0610
10 Number of Scans 32
11 Receiver Gain 106
12 Relaxation Delay 1.0000
13 Pulse Width 15.0000
14 Presaturation Frequency
15 Acquisition Time 4.0894
16 Acquisition Date 2018-10-30T21:45:00
17 Modification Date 2018-10-30T21:45:09
18 Spectrometer Frequency 400.19
19 Spectral Width 8012.8
20 Lowest Frequency -1535.1
21 Nucleus 1H
22 Acquired Size 32768
23 Spectral Size 65536
7.815
7.800
7.795
7.553
7.549
7.545
7.537
7.531
7.527
7.240
7.220
7.009
6.991
6.988
6.959
6.944
6.938
5.588
5.577
5.558
5.547
3.894
3.864
3.848
3.818
3.472
3.380
3.352 HDO
3.197
3.185
3.177
3.151
3.140
2.521 DMSO
2.516 DMSO
2.512 DMSO
2.507 DMSO
2.313
2.08
4.10
2.08
2.02
2.00
1.00
1.04
1.05
16 15 14 13 12 11 10 9 8 7 6 5 4 3 2 1 0 -1 -2 -3
f1 (ppm)
Br
O
H3C
N
N
O
Cl

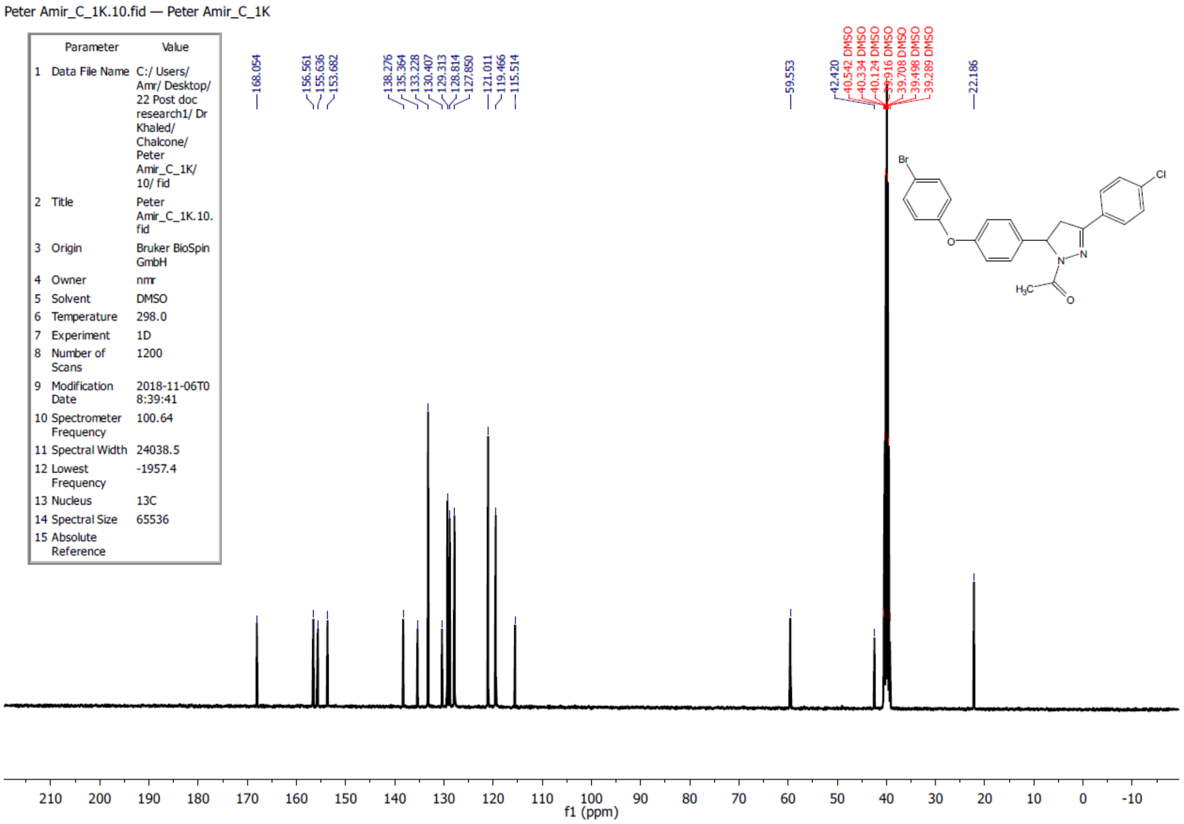
Peter Amir_C_1K.10.fid — Peter Amir_C_1K
Parameter Value
1 Data File Name C:/ Users/ Amr/ Desktop/ 22 Post doc research1/ Dr Khaled/ Chalcone/ Peter Amir_C_1K/ 10/ fid
2 Title Peter Amir_C_1K.10.fid
3 Origin Bruker BioSpin GmbH
4 Owner nmr
5 Solvent DMSO
6 Temperature 298.0
7 Experiment 1D
8 Number of Scans 1200
9 Modification Date 2018-11-06T08:39:41
10 Spectrometer Frequency 100.64
11 Spectral Width 24038.5
12 Lowest Frequency -1957.4
13 Nucleus 13C
14 Spectral Size 65536
15 Absolute Reference
168.054
156.561
155.636
153.682
138.276
135.364
133.228
130.407
129.313
128.814
127.850
121.011
119.466
115.514
59.553
42.420
40.542 DMSO
40.334 DMSO
40.124 DMSO
39.916 DMSO
39.708 DMSO
39.498 DMSO
39.289 DMSO
22.186
210 200 190 180 170 160 150 140 130 120 110 100 90 80 70 60 50 40 30 20 10 0 -10
f1 (ppm)
Br
O
Cl
N
N
H3C
O

Compound 3b

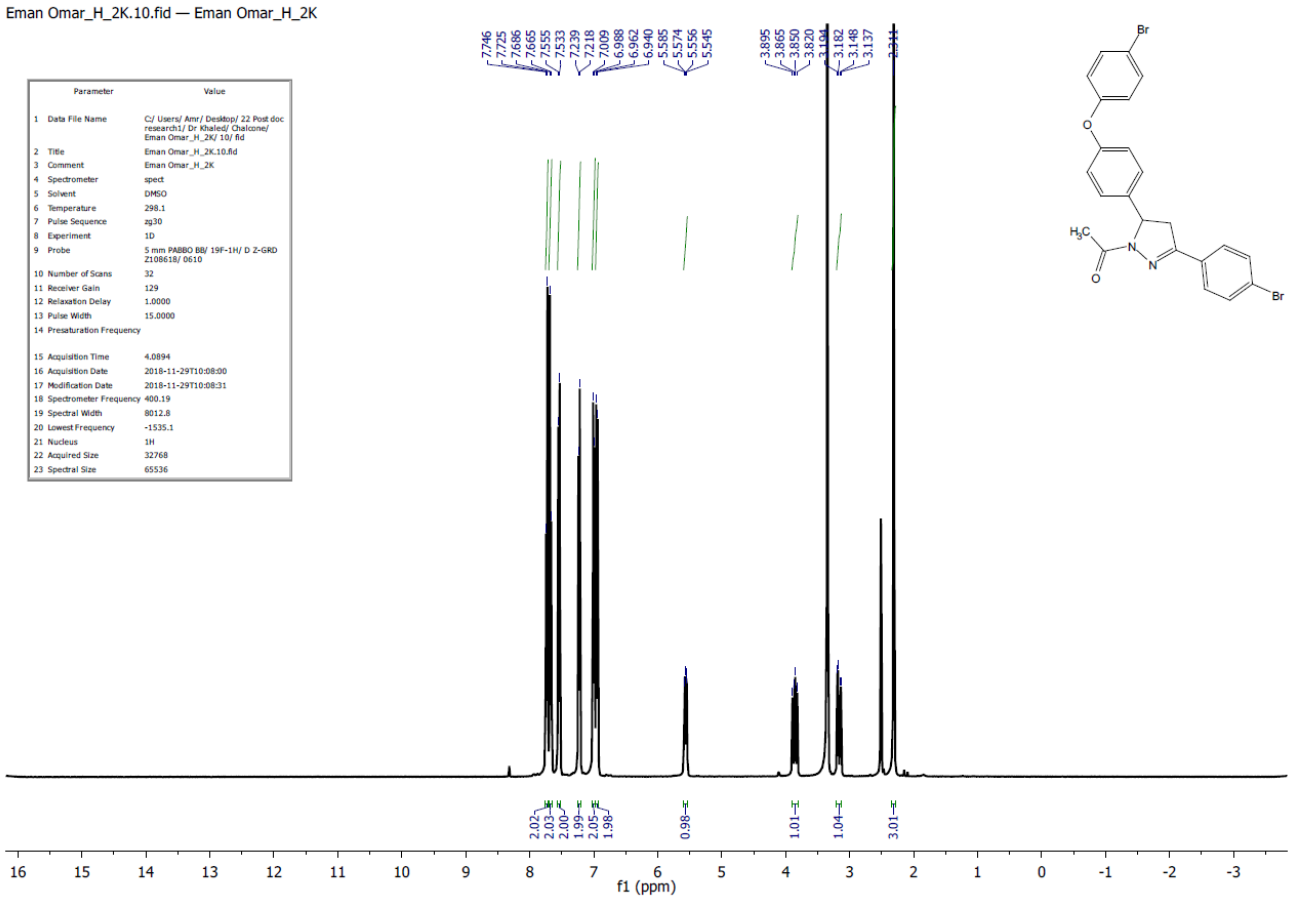

Eman Omar_H_2K.10.fid — Eman Omar_H_2K
Parameter Value
1 Data File Name C:/ Users/ Amr/ Desktop/ 22 Post doc research1/ Dr Khaled/ Chalcone/ Eman Omar_H_2K/ 10/ fid
2 Title Eman Omar_H_2K.10.fid
3 Comment Eman Omar_H_2K
4 Spectrometer spect
5 Solvent DMSO
6 Temperature 298.1
7 Pulse Sequence zg30
8 Experiment 1D
9 Probe 5 mm PABBO BB/ 19F-1H/ D Z-GRD Z108618/ 0610
10 Number of Scans 32
11 Receiver Gain 129
12 Relaxation Delay 1.0000
13 Pulse Width 15.0000
14 Presaturation Frequency
15 Acquisition Time 4.0894
16 Acquisition Date 2018-11-29T10:08:00
17 Modification Date 2018-11-29T10:08:31
18 Spectrometer Frequency 400.19
19 Spectral Width 8012.8
20 Lowest Frequency -1535.1
21 Nucleus 1H
22 Acquired Size 32768
23 Spectral Size 65536
7.746 7.725 7.686 7.665 7.555 7.533 7.239 7.218 7.009 6.988 6.962 6.940 5.585 5.574 5.556 5.545
3.895 3.865 3.850 3.820 3.194 3.182 3.148 3.137 2.311
2.02 2.03 2.00 1.99 2.05 1.98 0.98 1.01 1.04 3.01
Br
O
H3C
N
N
O
Br
f1 (ppm)

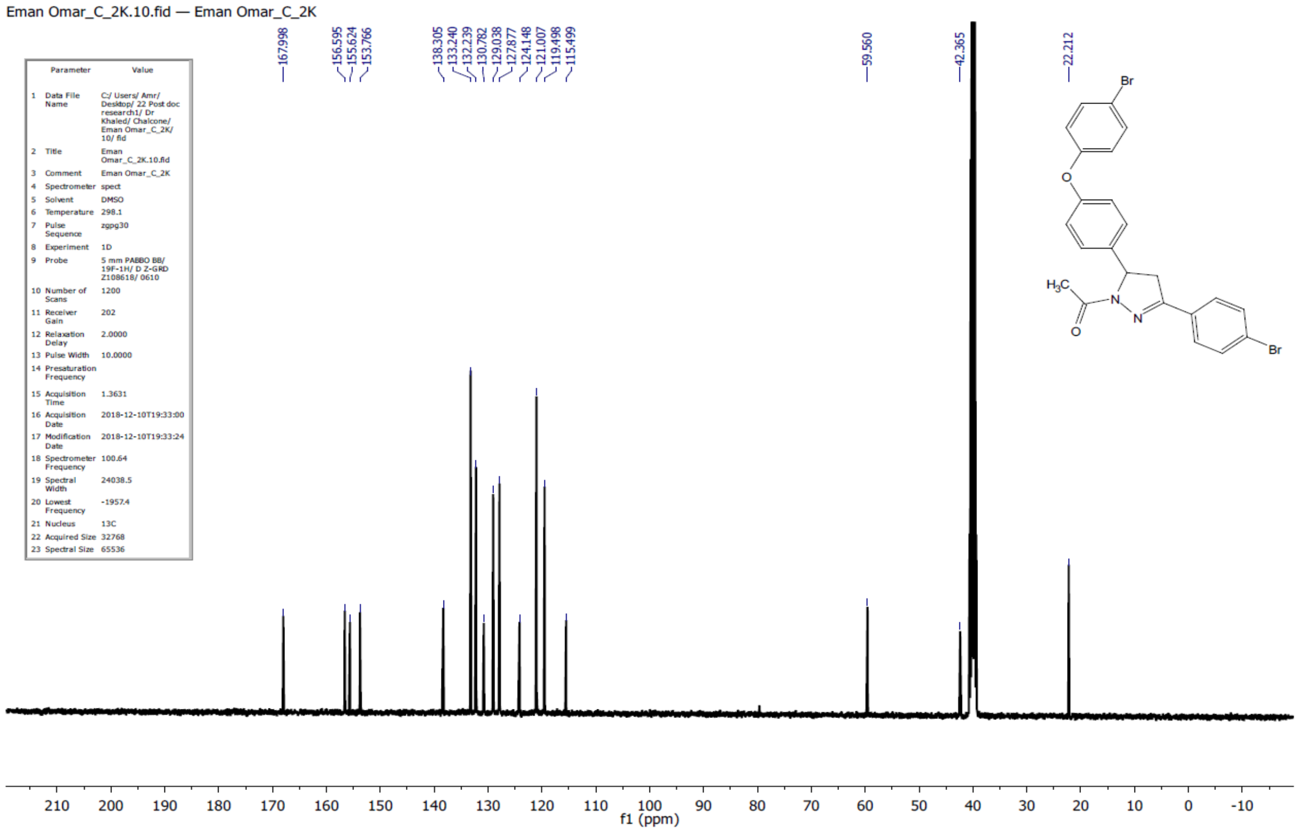

Eman Omar_C_2K.10.fid — Eman Omar_C_2K
Parameter Value
1 Data File Name C:/ Users/ Amr/ Desktop/ 22 Post doc research1/ Dr Khaled/ Chalcone/ Eman Omar_C_2K/ 10/ fid
2 Title Eman Omar_C_2K.10.fid
3 Comment Eman Omar_C_2K
4 Spectrometer spect
5 Solvent DMSO
6 Temperature 298.1
7 Pulse Sequence zgpg30
8 Experiment 1D
9 Probe 5 mm PABBO BB/ 19F-1H/ D Z-GRD Z108618/ 0610
10 Number of Scans 1200
11 Receiver Gain 202
12 Relaxation Delay 2.0000
13 Pulse Width 10.0000
14 Presaturation Frequency
15 Acquisition Time 1.3631
16 Acquisition Date 2018-12-10T19:33:00
17 Modification Date 2018-12-10T19:33:24
18 Spectrometer Frequency 100.64
19 Spectral Width 24038.5
20 Lowest Frequency -1957.4
21 Nucleus 13C
22 Acquired Size 32768
23 Spectral Size 65536
167.998 156.595 155.624 153.766 138.305 133.240 132.239 130.782 129.038 127.877 124.148 121.007 119.498 115.499 59.560 42.365 22.212
Br
O
H3C
N
N
O
Br
f1 (ppm)

Compound 3c

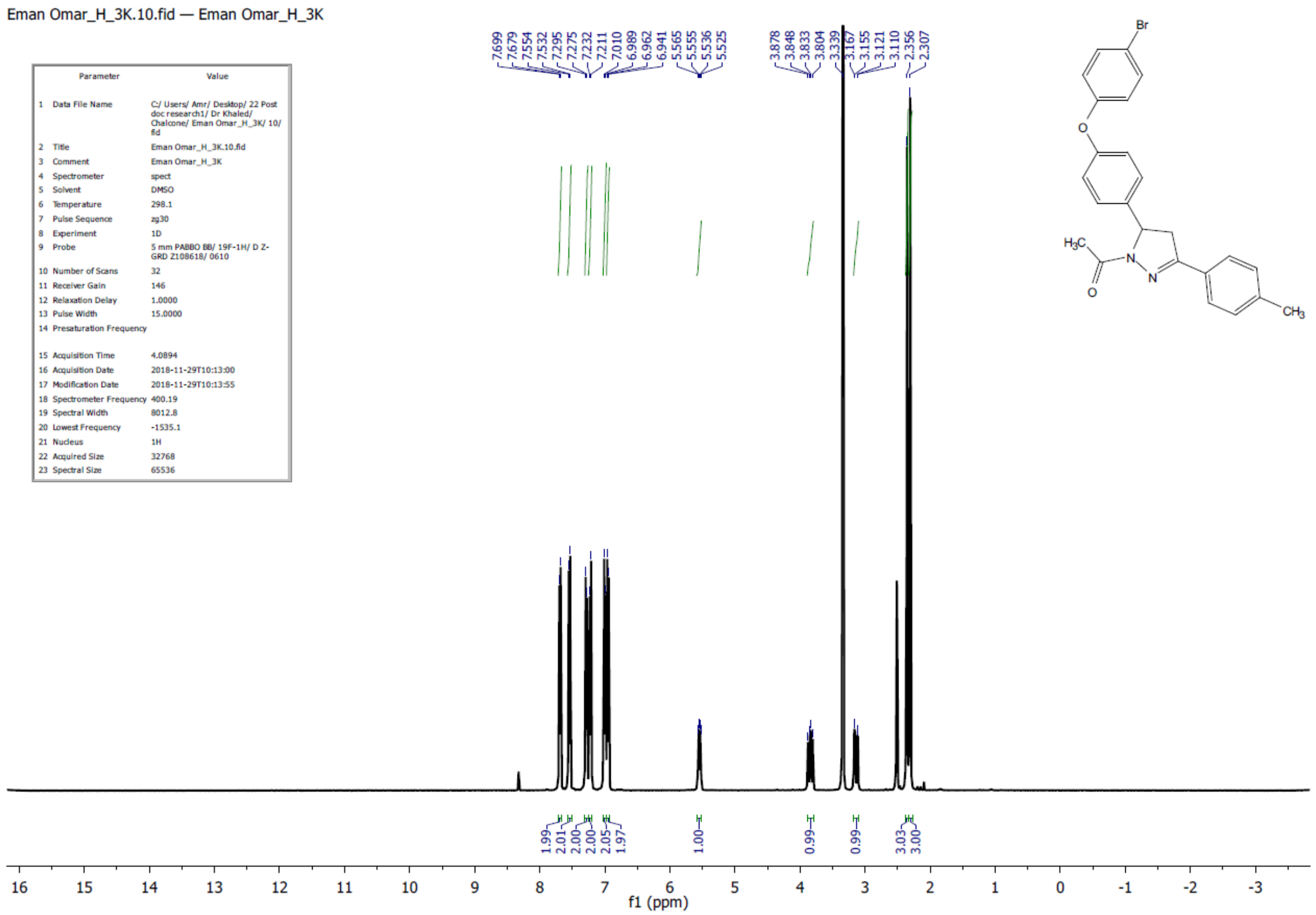
Eman Omar_H_3K.10.fid — Eman Omar_H_3K
Parameter | Value
1 Data File Name | C:/ Users/ Amr/ Desktop/ 22 Post doc research1/ Dr Khaled/ Chalcone/ Eman Omar_H_3K/ 10/ fid
2 Title | Eman Omar_H_3K.10.fid
3 Comment | Eman Omar_H_3K
4 Spectrometer | spect
5 Solvent | DMSO
6 Temperature | 298.1
7 Pulse Sequence | zg30
8 Experiment | 1D
9 Probe | 5 mm PABBO BB/ 19F-1H/ D Z-GRD Z108618/ 0610
10 Number of Scans | 32
11 Receiver Gain | 146
12 Relaxation Delay | 1.0000
13 Pulse Width | 15.0000
14 Presaturation Frequency
15 Acquisition Time | 4.0894
16 Acquisition Date | 2018-11-29T10:13:00
17 Modification Date | 2018-11-29T10:13:55
18 Spectrometer Frequency | 400.19
19 Spectral Width | 8012.8
20 Lowest Frequency | -1535.1
21 Nucleus | 1H
22 Acquired Size | 32768
23 Spectral Size | 65536
7.699 7.679 7.554 7.532 7.295 7.275 7.232 7.211 7.010 6.989 6.962 6.941 5.565 5.555 5.536 5.525 3.878 3.848 3.833 3.804 3.339 3.167 3.155 3.121 3.110 2.356 2.307
1.99 2.01 2.00 2.00 2.05 1.97 1.00 0.99 0.99 3.03 3.00
f1 (ppm)
Br
O
H3C
N
N
O
CH3

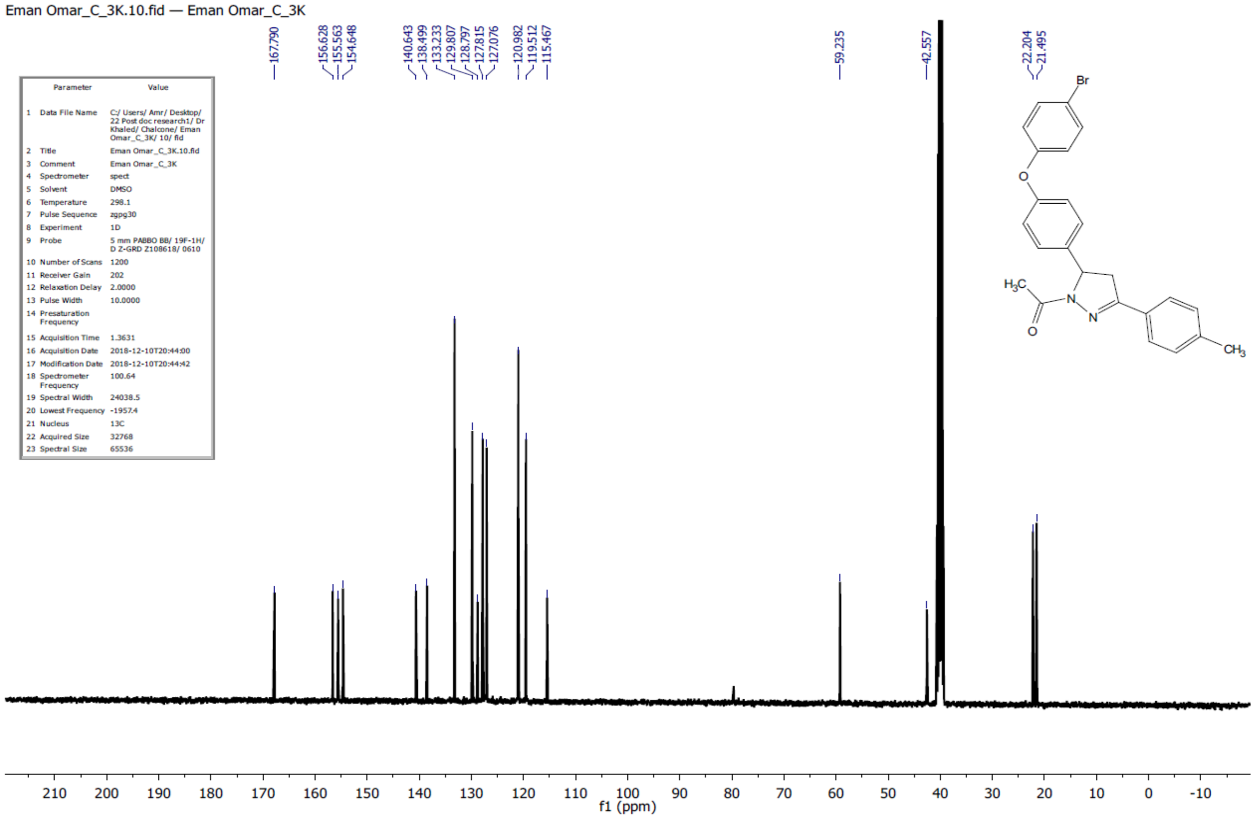
Eman Omar_C_3K.10.fid — Eman Omar_C_3K
Parameter | Value
1 Data File Name | C:/ Users/ Amr/ Desktop/ 22 Post doc research1/ Dr Khaled/ Chalcone/ Eman Omar_C_3K/ 10/ fid
2 Title | Eman Omar_C_3K.10.fid
3 Comment | Eman Omar_C_3K
4 Spectrometer | spect
5 Solvent | DMSO
6 Temperature | 298.1
7 Pulse Sequence | zgpg30
8 Experiment | 1D
9 Probe | 5 mm PABBO BB/ 19F-1H/ D Z-GRD Z108618/ 0610
10 Number of Scans | 1200
11 Receiver Gain | 202
12 Relaxation Delay | 2.0000
13 Pulse Width | 10.0000
14 Presaturation Frequency
15 Acquisition Time | 1.3631
16 Acquisition Date | 2018-12-10T20:44:00
17 Modification Date | 2018-12-10T20:44:42
18 Spectrometer Frequency | 100.64
19 Spectral Width | 24038.5
20 Lowest Frequency | -1957.4
21 Nucleus | 13C
22 Acquired Size | 32768
23 Spectral Size | 65536
167.790 156.628 155.563 154.648 140.643 138.499 133.233 129.807 128.797 127.815 127.076 120.982 119.512 115.467 59.235 42.557 22.204 21.495
f1 (ppm)
Br
O
H3C
N
N
O
CH3

Compound 3d

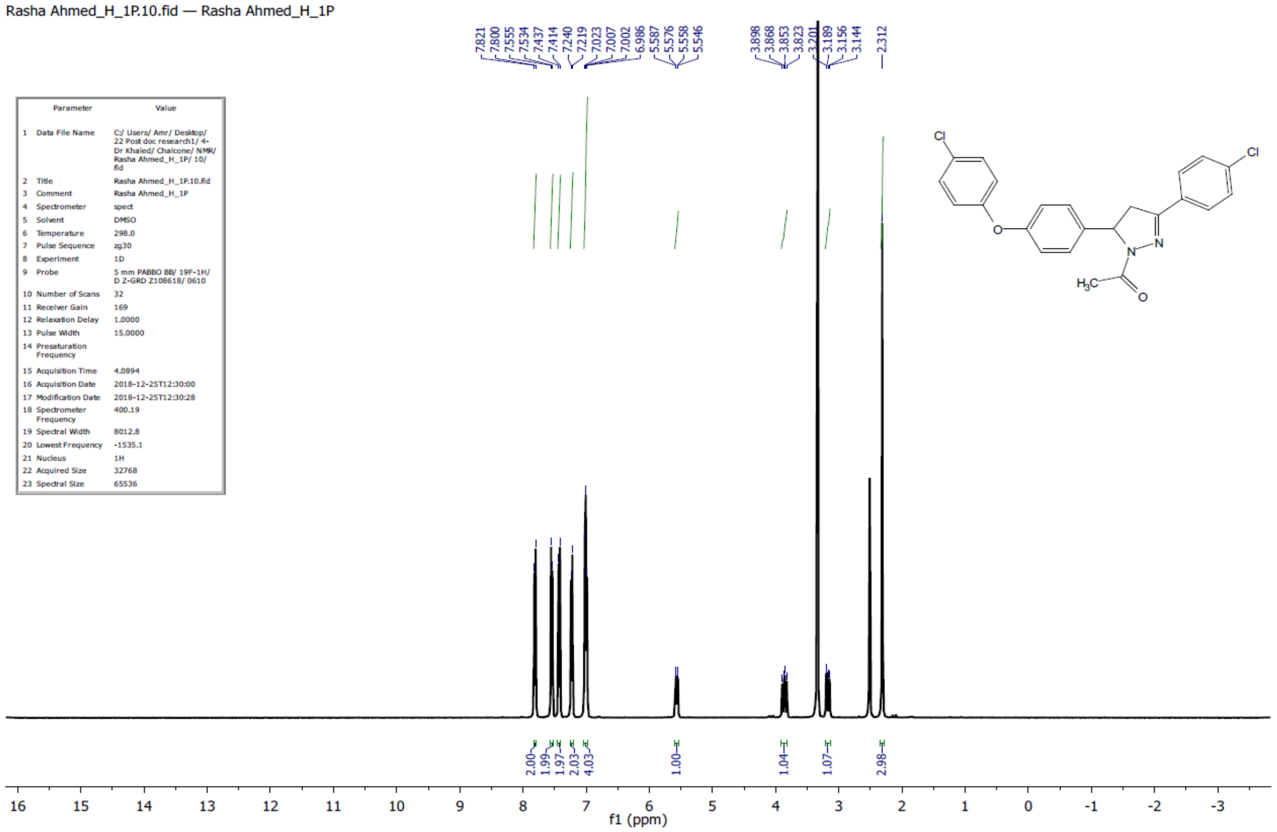
Rasha Ahmed_H_1P.10.fid — Rasha Ahmed_H_1P
7.821
7.800
7.555
7.534
7.437
7.414
7.240
7.219
7.023
7.007
7.002
6.986
5.587
5.576
5.558
5.546
3.898
3.868
3.853
3.823
3.201
3.189
3.156
3.144
2.312
Parameter Value
1 Data File Name C:/ Users/ Amr/ Desktop/ 22 Post doc research1/ 4- Dr Khaled/ Chalcone/ NMR/ Rasha Ahmed_H_1P/ 10/ fid
2 Title Rasha Ahmed_H_1P.10.fid
3 Comment Rasha Ahmed_H_1P
4 Spectrometer spect
5 Solvent DMSO
6 Temperature 298.0
7 Pulse Sequence zg30
8 Experiment 1D
9 Probe 5 mm PABBO BB/ 19F-1H/ D Z-GRD Z108618/ 0610
10 Number of Scans 32
11 Receiver Gain 169
12 Relaxation Delay 1.0000
13 Pulse Width 15.0000
14 Presaturation Frequency
15 Acquisition Time 4.0894
16 Acquisition Date 2018-12-25T12:30:00
17 Modification Date 2018-12-25T12:30:28
18 Spectrometer Frequency 400.19
19 Spectral Width 8012.8
20 Lowest Frequency -1535.1
21 Nucleus 1H
22 Acquired Size 32768
23 Spectral Size 65536
Cl
Cl
O
N
N
H3C
O
2.00
1.99
1.97
2.03
4.03
1.00
1.04
1.07
2.98
16 15 14 13 12 11 10 9 8 7 6 5 4 3 2 1 0 -1 -2 -3
f1 (ppm)

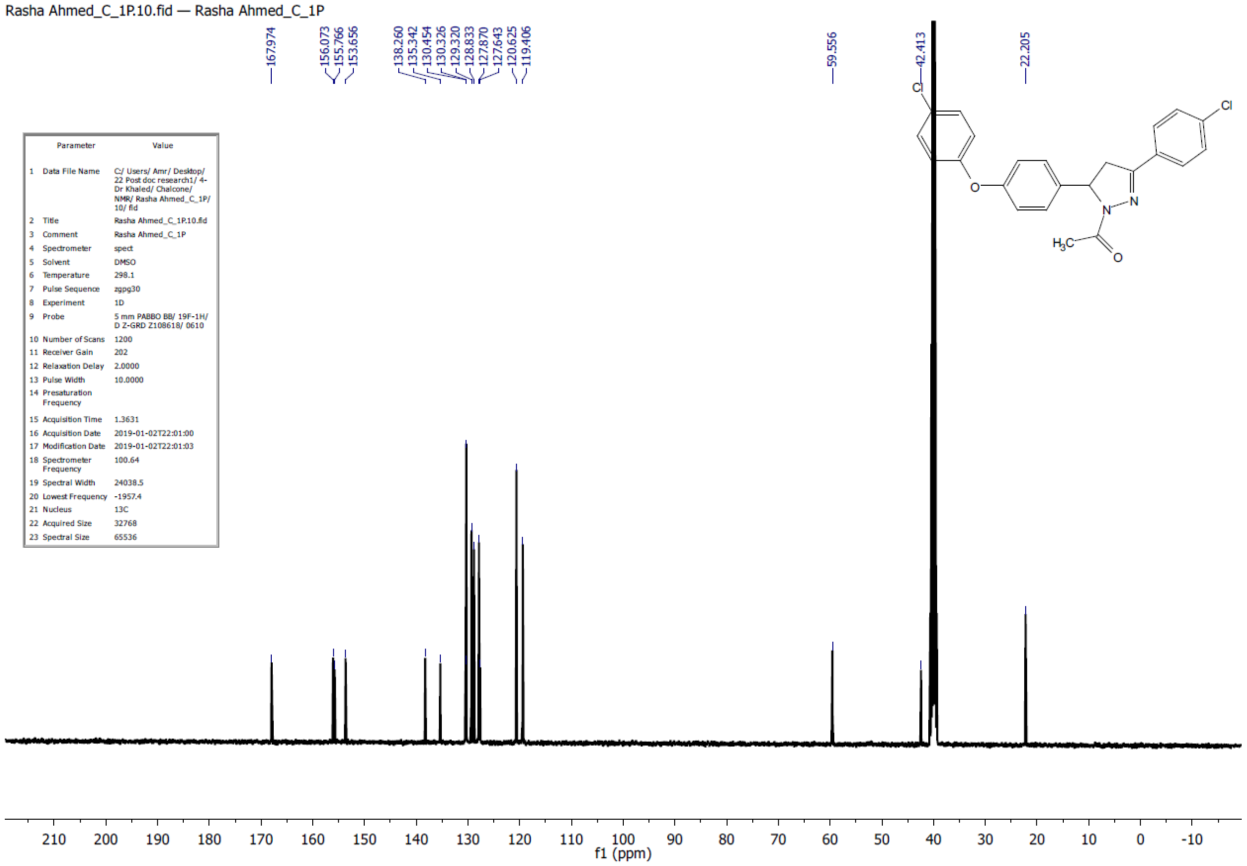
Rasha Ahmed_C_1P.10.fid — Rasha Ahmed_C_1P
167.974
156.073
155.766
153.656
138.260
135.342
130.454
130.326
129.320
128.833
127.870
127.643
120.625
119.406
59.556
42.413
22.205
Parameter Value
1 Data File Name C:/ Users/ Amr/ Desktop/ 22 Post doc research1/ 4- Dr Khaled/ Chalcone/ NMR/ Rasha Ahmed_C_1P/ 10/ fid
2 Title Rasha Ahmed_C_1P.10.fid
3 Comment Rasha Ahmed_C_1P
4 Spectrometer spect
5 Solvent DMSO
6 Temperature 298.1
7 Pulse Sequence zgpg30
8 Experiment 1D
9 Probe 5 mm PABBO BB/ 19F-1H/ D Z-GRD Z108618/ 0610
10 Number of Scans 1200
11 Receiver Gain 202
12 Relaxation Delay 2.0000
13 Pulse Width 10.0000
14 Presaturation Frequency
15 Acquisition Time 1.3631
16 Acquisition Date 2019-01-02T22:01:00
17 Modification Date 2019-01-02T22:01:03
18 Spectrometer Frequency 100.64
19 Spectral Width 24038.5
20 Lowest Frequency -1957.4
21 Nucleus 13C
22 Acquired Size 32768
23 Spectral Size 65536
Cl
Cl
O
N
N
H3C
O
210 200 190 180 170 160 150 140 130 120 110 100 90 80 70 60 50 40 30 20 10 0 -10
f1 (ppm)

Compound 3e

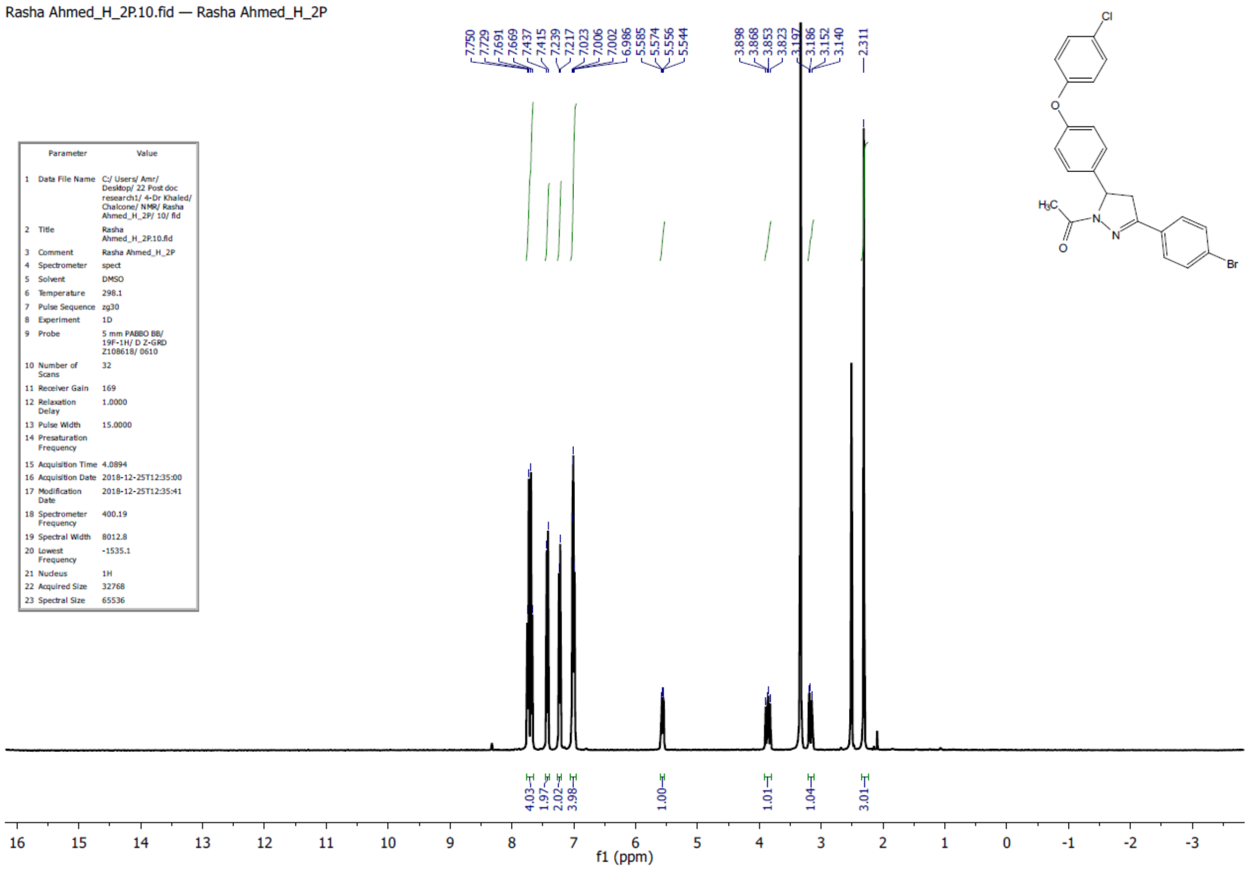
Rasha Ahmed_H_2P.10.fid — Rasha Ahmed_H_2P
7.750 7.729 7.691 7.669 7.437 7.415 7.239 7.217 7.023 7.006 7.002 6.986 5.585 5.574 5.556 5.544 3.898 3.868 3.853 3.823 3.197 3.186 3.152 3.140 2.311
Parameter Value
1 Data File Name C:/ Users/ Amr/ Desktop/ 22 Post doc research1/ 4-Dr Khaled/ Chalcone/ NMR/ Rasha Ahmed_H_2P/ 10/ fid
2 Title Rasha Ahmed_H_2P.10.fid
3 Comment Rasha Ahmed_H_2P
4 Spectrometer spect
5 Solvent DMSO
6 Temperature 298.1
7 Pulse Sequence zg30
8 Experiment 1D
9 Probe 5 mm PABBO BB/ 19F-1H/ D Z-GRD Z108618/ 0610
10 Number of Scans 32
11 Receiver Gain 169
12 Relaxation Delay 1.0000
13 Pulse Width 15.0000
14 Presaturation Frequency
15 Acquisition Time 4.0894
16 Acquisition Date 2018-12-25T12:35:00
17 Modification Date 2018-12-25T12:35:41
18 Spectrometer Frequency 400.19
19 Spectral Width 8012.8
20 Lowest Frequency -1535.1
21 Nucleus 1H
22 Acquired Size 32768
23 Spectral Size 65536
Cl
O
H3C
N
N
O
Br
4.03 1.97 2.02 3.98 1.00 1.01 1.04 3.01
16 15 14 13 12 11 10 9 8 7 6 5 4 3 2 1 0 -1 -2 -3
f1 (ppm)

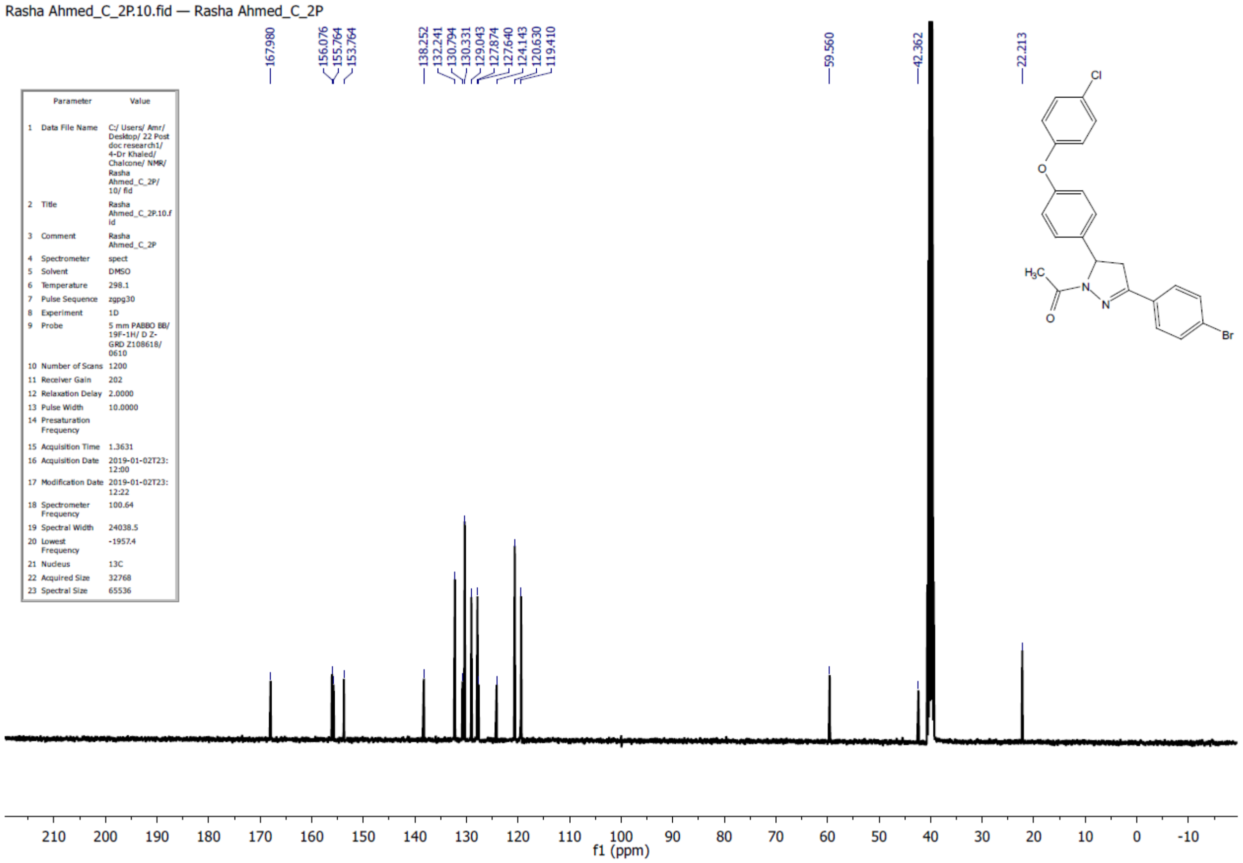
Rasha Ahmed_C_2P.10.fid — Rasha Ahmed_C_2P
167.980 156.076 155.764 153.764 138.252 132.241 130.794 130.331 129.043 127.874 127.640 124.143 120.630 119.410 59.560 42.362 22.213
Parameter Value
1 Data File Name C:/ Users/ Amr/ Desktop/ 22 Post doc research1/ 4-Dr Khaled/ Chalcone/ NMR/ Rasha Ahmed_C_2P/ 10/ fid
2 Title Rasha Ahmed_C_2P.10.fid
3 Comment Rasha Ahmed_C_2P
4 Spectrometer spect
5 Solvent DMSO
6 Temperature 298.1
7 Pulse Sequence zgpg30
8 Experiment 1D
9 Probe 5 mm PABBO BB/ 19F-1H/ D Z-GRD Z108618/ 0610
10 Number of Scans 1200
11 Receiver Gain 202
12 Relaxation Delay 2.0000
13 Pulse Width 10.0000
14 Presaturation Frequency
15 Acquisition Time 1.3631
16 Acquisition Date 2019-01-02T23:12:00
17 Modification Date 2019-01-02T23:12:22
18 Spectrometer Frequency 100.64
19 Spectral Width 24038.5
20 Lowest Frequency -1957.4
21 Nucleus 13C
22 Acquired Size 32768
23 Spectral Size 65536
Cl
O
H3C
N
N
O
Br
210 200 190 180 170 160 150 140 130 120 110 100 90 80 70 60 50 40 30 20 10 0 -10
f1 (ppm)

Compound 3f

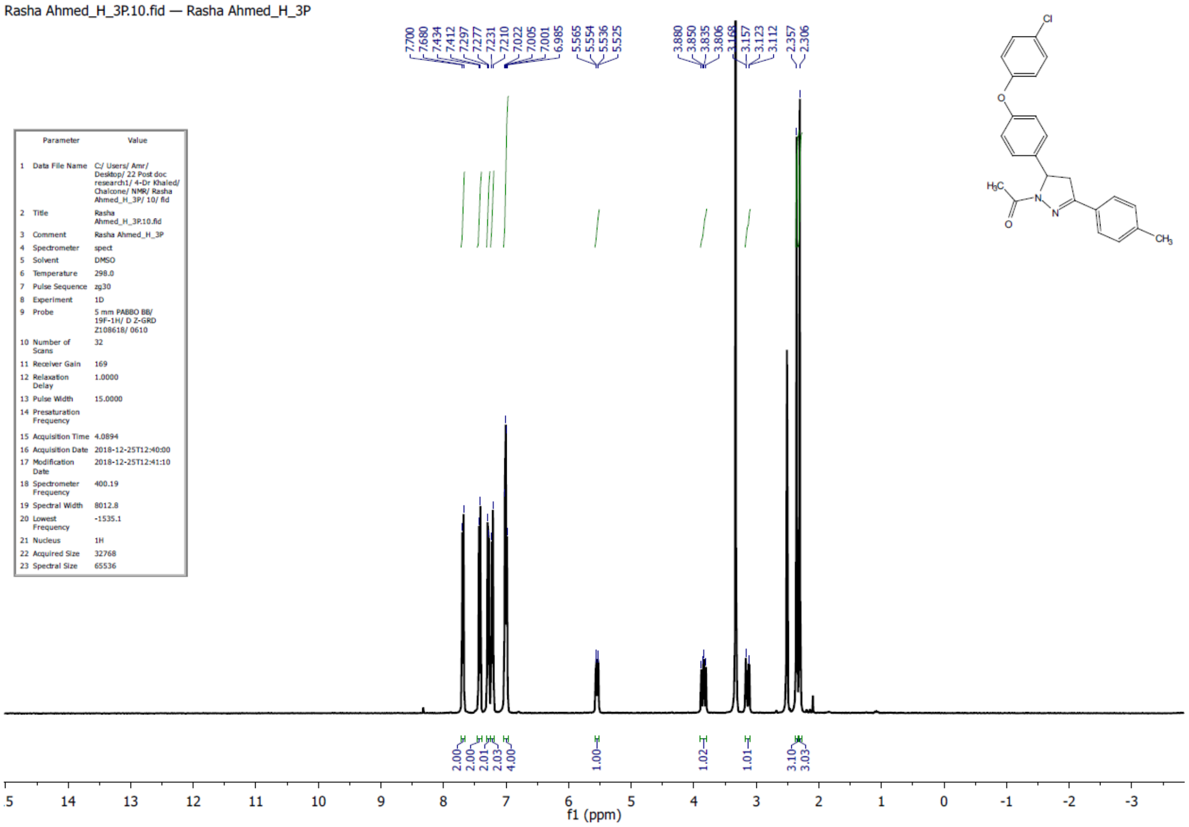
Rasha Ahmed_H_3P.10.fid — Rasha Ahmed_H_3P
7.700 7.680 7.434 7.412 7.297 7.277 7.231 7.210 7.022 7.005 7.001 6.985
5.565 5.554 5.536 5.525
3.880 3.850 3.835 3.806 3.168 3.157 3.123 3.112
2.357 2.306
Parameter Value
1 Data File Name C:/ Users/ Amr/ Desktop/ 22 Post doc research1/ 4-Dr Khaled/ Chalcone/ NMR/ Rasha Ahmed_H_3P/ 10/ fid
2 Title Rasha Ahmed_H_3P.10.fid
3 Comment Rasha Ahmed_H_3P
4 Spectrometer spect
5 Solvent DMSO
6 Temperature 298.0
7 Pulse Sequence zg30
8 Experiment 1D
9 Probe 5 mm PABBO BB/ 19F-1H/ D Z-GRD Z108618/ 0610
10 Number of Scans 32
11 Receiver Gain 169
12 Relaxation Delay 1.0000
13 Pulse Width 15.0000
14 Presaturation Frequency
15 Acquisition Time 4.0894
16 Acquisition Date 2018-12-25T12:40:00
17 Modification Date 2018-12-25T12:41:10
18 Spectrometer Frequency 400.19
19 Spectral Width 8012.8
20 Lowest Frequency -1535.1
21 Nucleus 1H
22 Acquired Size 32768
23 Spectral Size 65536
Cl
O
H3C
N
N
O
CH3
2.00 2.00 2.01 2.03 4.00
1.00
1.02
1.01
3.10 3.03
5 14 13 12 11 10 9 8 7 6 5 4 3 2 1 0 -1 -2 -3
f1 (ppm)

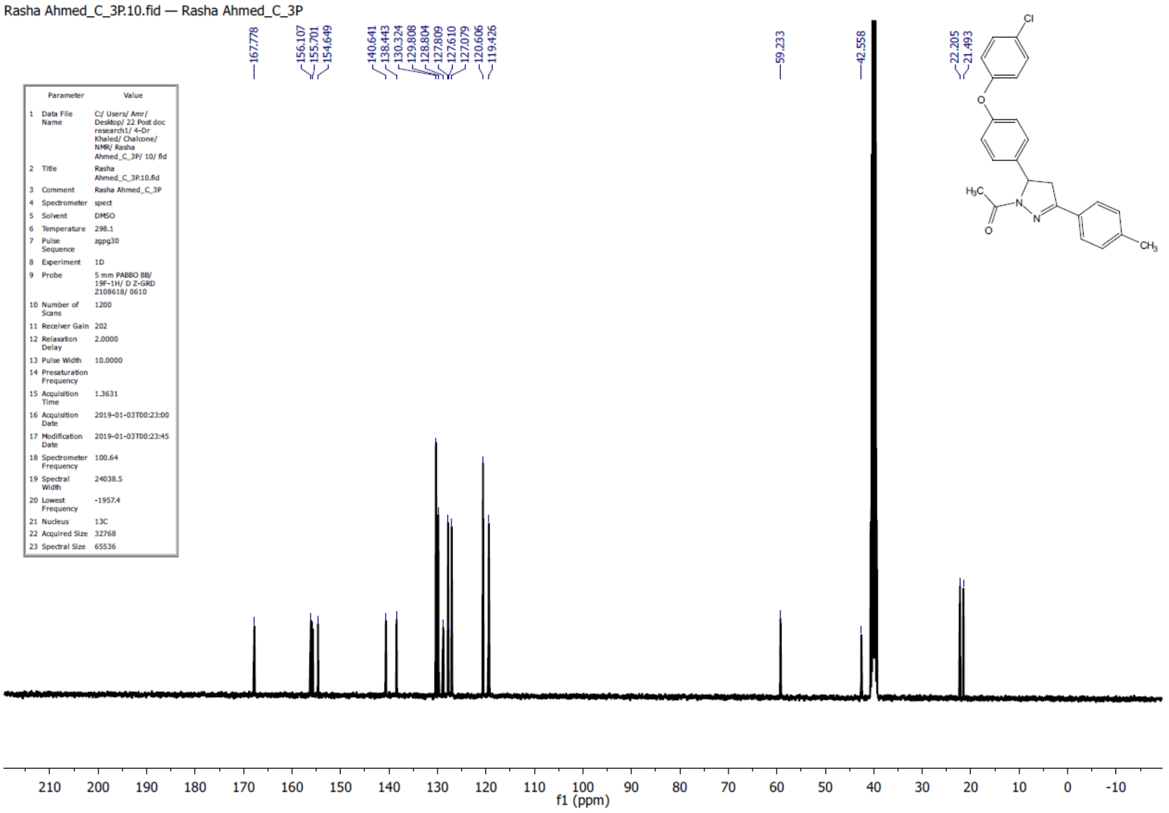
Rasha Ahmed_C_3P.10.fid — Rasha Ahmed_C_3P
167.778
156.107 155.701 154.649
140.641 138.443 130.324 129.808 128.804 127.809 127.610 127.079 120.606 119.426
59.233
42.558
22.205 21.493
Parameter Value
1 Data File Name C:/ Users/ Amr/ Desktop/ 22 Post doc research1/ 4-Dr Khaled/ Chalcone/ NMR/ Rasha Ahmed_C_3P/ 10/ fid
2 Title Rasha Ahmed_C_3P.10.fid
3 Comment Rasha Ahmed_C_3P
4 Spectrometer spect
5 Solvent DMSO
6 Temperature 298.1
7 Pulse Sequence zgpg30
8 Experiment 1D
9 Probe 5 mm PABBO BB/ 19F-1H/ D Z-GRD Z108618/ 0610
10 Number of Scans 1200
11 Receiver Gain 202
12 Relaxation Delay 2.0000
13 Pulse Width 10.0000
14 Presaturation Frequency
15 Acquisition Time 1.3631
16 Acquisition Date 2019-01-03T00:23:00
17 Modification Date 2019-01-03T00:23:45
18 Spectrometer Frequency 100.64
19 Spectral Width 24038.5
20 Lowest Frequency -1957.4
21 Nucleus 13C
22 Acquired Size 32768
23 Spectral Size 65536
Cl
O
H3C
N
N
O
CH3
210 200 190 180 170 160 150 140 130 120 110 100 90 80 70 60 50 40 30 20 10 0 -10
f1 (ppm)
